# Review of the Integrated Approaches for Monitoring and Treating Parabens in Water Matrices

**DOI:** 10.3390/molecules29235533

**Published:** 2024-11-22

**Authors:** Denga Ramutshatsha-Makhwedzha, Tshimangadzo S. Munonde

**Affiliations:** Institute for Nanotechnology and Water Sustainability, College of Science, Engineering, and Technology, University of South Africa, Florida Science Campus, Roodepoort 1710, South Africa

**Keywords:** parabens, wastewater, sample preparation, adsorption, degradation, endocrine disruptors

## Abstract

Due to their antibacterial and antifungal properties, parabens are commonly used as biocides and preservatives in food, cosmetics, and pharmaceuticals. Parabens have been reported to exist in various water matrices at low concentrations, which renders the need for sample preparation before their quantification using analytical techniques. Thus, sample preparation methods such as solid-phase extraction (SPE), rotating-disk sorptive extraction (RDSE), and vortex-assisted dispersive liquid–liquid extraction (VA-DLLE) that are commonly used for parabens extraction and preconcentration have been discussed. As a result of sample preparation methods, analytical techniques now detect parabens at trace levels ranging from µg/L to ng/L. These compounds have been detected in water, air, soil, and human tissues. While the full impact of parabens on human health and ecosystems is still being debated in the scientific community, it is widely recognized that parabens can act as endocrine disruptors. Furthermore, some studies have suggested that parabens may have carcinogenic effects. The presence of parabens in the environment is primarily due to wastewater discharges, which result in widespread contamination and their concentrations increased during the COVID-19 pandemic waves. Neglecting the presence of parabens in water exposes humans to these compounds through contaminated food and drinking water. Although there are reviews that focus on the occurrence, fate, and behavior of parabens in the environment, they frequently overlook critical aspects such as removal methods, policy development, and regulatory frameworks. Addressing this gap, the effective treatment of parabens in water relies on combined approaches that address both cost and operational challenges. Membrane filtration methods, such as nanofiltration (NF) and reverse osmosis (RO), demonstrate high efficacy but are hindered by maintenance and energy costs due to extensive fouling. Innovations in anti-fouling and energy efficiency, coupled with pre-treatment methods like adsorption, help mitigate these costs and enhance scalability. Furthermore, combining adsorption with advanced oxidation processes (AOPs) or biological treatments significantly improves economic and energy efficiency. Integrating systems like O₃/UV with activated carbon, along with byproduct recovery strategies, further advances circular economy goals by minimizing waste and resource use. This review provides a thorough overview of paraben monitoring in wastewater, current treatment techniques, and the regulatory policies that govern their presence. Furthermore, it provides perspectives that are critical for future scientific investigations and shaping policies aimed at mitigating the risks of parabens in drinking water.

## 1. Introduction

In recent decades, research has highlighted water pollution from synthetic organic compounds as a significant threat to both environmental and public health. The growing global population and industrial and agricultural expansion have significantly contributed to an increase in organic chemical pollution [1]. The release of large amounts of wastewater containing organic and inorganic chemicals and complex compounds strains water bodies, degrading their quality below the environment’s natural resilience and tolerance [2]. Among the most pervasive contaminants in aquatic systems are parabens—synthetic preservatives found widely in cosmetics, pharmaceuticals, food products, and personal care items. Parabens, such as methylparaben, ethylparaben, propylparaben, and butylparaben, are valued for their antibacterial and antifungal properties, which enhance the shelf life of various products [3,4]. Yet, they have been classified as “emerging pollutants” due to their persistence in water matrices, potential bioaccumulation, and capacity to disrupt biological processes in both humans and aquatic organisms [5].

The US Environmental Protection Agency classifies parabens as endocrine-disrupting pollutants (EDCs) [6]. Parabens can disrupt the endocrine system, thereby harming the human body. Given their limited removal in conventional wastewater treatment processes, parabens continuously enter water bodies, intensifying the urgency to develop effective monitoring and treatment techniques that mitigate their environmental impact. This serious global issue demands rapid monitoring and adoption of quality control measures [3]. The toxicological effects of parabens underscore the need for careful monitoring and management. Studies indicate that parabens can interfere with endocrine functions by mimicking hormones like estrogen, disrupting hormonal balance even at low concentrations [7,8,9]. This disruption affects the reproductive and developmental health of aquatic species and can impact higher organisms through bioaccumulation [10]. Additional research suggests that parabens may induce genotoxicity, oxidative stress, and immunotoxicity, posing further risks to human health [11,12]. These toxicological findings emphasize the importance of effective monitoring and treatment strategies to reduce paraben contamination in water matrices. The environmental impact of parabens extends beyond toxicity. Once released into water systems, they undergo complex interactions that can lead to more harmful byproducts. For example, exposure to ultraviolet (UV) light and the presence of specific minerals can degrade parabens into compounds like p-hydroxybenzoic acid, which itself can harm ecosystems [13]. The persistence of parabens and their byproducts, particularly in low-circulation environments, can cause cumulative damage, raising concerns as industrial activities and urbanization increase the volume of parabens released into natural water bodies [5]. Consequently, developing cost-effective, sustainable treatment methods tailored for paraben removal in diverse water matrices is critical. Recently, research has focused on integrated approaches that combine multiple technologies, such as advanced oxidation processes (AOPs), membrane filtration, and adsorption, to enhance removal efficiency [14]. Traditional wastewater treatment plants (WWTPs) are often ineffective in removing emerging contaminants such as parabens [15]. Common processes in WWTPs, such as coagulation [16], sedimentation, and basic filtration, typically fall short of fully eliminating trace contaminants, especially those with moderate to high hydrophobicity and low biodegradability [17]. Advanced methods—such as activated carbon adsorption, membrane filtration, and AOPs—are effective for organic micropollutant removal but are often costly and energy-intensive, limiting their application on a broad scale [16,18].

Lee et al. [16] investigated the reaction kinetics and degradation efficiency of halogenated methylparaben during ozonation and UV/H_2_O_2_ treatment of drinking water and wastewater effluent. Results show that in simulated treatments of drinking water and wastewater effluent, parabens were efficiently eliminated during ozonation, requiring a specific ozone dose of >0.26 gO_3_/g DOC for over 97% degradation. UV/H_2_O_2_ treatment with 10 mg/L H_2_O_2_ resulted in over 90% degradation at a UV fluence of 2000 mJ/cm^2^. Overall, ozonation and UV/H_2_O_2_ were effective in reducing parabens and their halogenated derivatives during advanced water treatment. Integrating multiple treatment methods has shown promise in improving paraben removal efficiency, making these integrated strategies potentially viable for large-scale implementation [14]. Advances in analytical methods now allow the detection of trace contaminants at parts-per-trillion levels, enabling more accurate and sensitive measurements of parabens in diverse water matrices. High-sensitivity techniques like liquid chromatography–mass spectrometry (LC–MS) and gas chromatography–mass spectrometry (GC–MS) have become standard for analyzing parabens due to their precision and reliability [19,20,21]. However, adapting these methods for rapid, on-site monitoring remains challenging. In recent years, interest in biosensors and portable detection devices has increased to achieve real-time paraben detection and facilitate more responsive water quality management [22]. Chen et al. [22] examined advanced sensor technologies for detecting contaminants in water, comparing the performance of biosensors, optical sensors, electrochemical sensors, and nanomaterial-based sensors. They recommend that all sensors undergo multi-point calibration, with quality control samples verified, spiked recovery experiments conducted, and system-level joint calibration completed to ensure accuracy. Additionally, a robust data management system is essential to maintain data integrity and traceability, using statistical analysis and data verification methods to detect anomalies.

Therefore, successful detection requires the extraction of parabens from complex aqueous matrices, which often contain multiple interfering substances. Several sample preparation methods have been developed to address this challenge. Techniques such as solid-phase microextraction [23], dispersive miniaturized solid-phase extraction (D-μSPE) [24], microwave-assisted extraction (MAE) [25], and liquid extraction [26] are commonly employed to isolate parabens from water samples. Each method offers unique advantages for enhancing detection sensitivity and specificity in paraben analysis, enabling more accurate quantification in various water environments.

The article’s main objectives include reviewing the most recent advances in monitoring technology, investigating alternative treatment strategies, and examining current policies and regulations of paraben levels around the world. This article seeks to provide a comprehensive overview of paraben monitoring and treatment in water, with a focus on integrated techniques. It begins by looking into improved monitoring tools that help detect parabens. This is followed by a thorough examination of several treatment strategies, including adsorption, a review of degradation methods, and ion exchange. The paper then addresses the integration of these strategies, emphasizing their potential to improve overall efficiency and effectiveness. It also examines the current policies and regulations governing paraben levels in water, evaluating their impact on monitoring and treatment options.

## 2. Sample Preparation Methods Combined with Analytical Techniques

**Figure 1:**
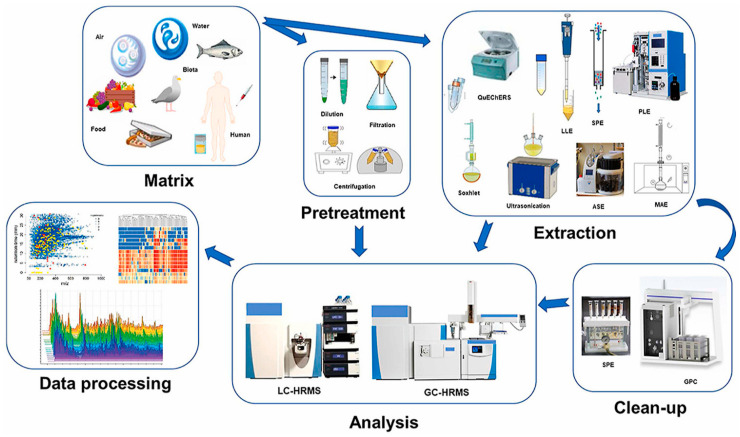
Step-by-step extraction and analysis of emerging contaminants. Reproduced with permission from [40].

The monitoring of parabens in drinking and wastewater is crucial due to their widespread use in personal care products, pharmaceuticals, and food, which leads to their presence in the aquatic environment [27,28,29]. Due to their use as preservatives, parabens such as ethylparaben (MetPB), ethylparaben (EtPB), butylparaben (BuPB) and propylparaben (PrPB), octylparaben (OctPB) and their derivatives, amongst others, have been shown to accumulate in wastewater systems and drinking water systems, causing potential endocrine disruption [29,30,31]. Several paraben compounds, as well as their metabolites, have been shown to have both oestrogenic and anti-androgenic effects in vitro and in vivo [32]. Due to this, parabens and their metabolites have been monitored in various matrices including surface water from rivers and dams, wastewater, groundwater, and drinking water [33]. However, parabens often exist in low quantities and are linked to the sample matrix; thus, analyzing them directly in complex matrices such as wastewater can be challenging [34,35]. Sample preparation is a crucial step in the analytical process required to enable the extraction, preconcentration, and sample clean-up via the removal of interfering substances and/or pre-concentration or enrichment of target analytes before their separation and quantification using chromatographic techniques [36,37,38], as demonstrated in Figure 1. This warrants that the paraben’s concentration levels are assessed at an appropriate concentration level [39].

Sample preparation is a critical step before analysis as it can significantly affect selectivity, sensitivity, and reproducibility. Figure 1 shows the critical steps involved in the pre-treatment, extraction and clean-up of the sample matrix to isolate the analytes of interest leading to high sensitivity during analysis with the analytical instrument of interest. Notably, the quality of the results obtained from the chromatographic analysis depends on the efficiency of the pre-treatment and extraction protocols employed. It is therefore important to choose a pre-treatment and extraction protocol that sufficiently cleans up the sample matrix and eliminates most unwanted interferences before chromatographic analysis. This should be done with a balance between sensitivity and minimizing the losses of compounds of interest.

Commonly used sample preparation techniques for parabens such as solid-phase extraction (SPE) [41], rotating-disk sorptive extraction (RDSE) [42], and vortex-assisted dispersive liquid–liquid extraction (VA-DLLE) [43], amongst others are discussed in this section. These sample preparation methods are integrated with analytical techniques in the monitoring of parabens in water [44,45,46]. To this end, several advanced analytical techniques are reported for identifying and quantifying parabens in water. These include liquid chromatography–tandem mass spectrometry (LC–MS/MS) [47], ultra-high performance liquid chromatography (UHPLC), coupled with time-of-flight mass spectrometry (TOF/MS) [48,49], gas chromatography–mass spectrometry (GC–MS) [50,51], and high-performance liquid chromatography (HPLC) with UV/DAD detection [52], amongst others. The following section will look at sample preparation techniques such as SPE, RDSE and VA-DLLE, amongst others, and their respective integrated chromatographic techniques selected based on their high sensitivity, specificity, polarity, detection limits and in some instances, the volatility of parabens.

### 2.1. Solid-Phase Extraction (SPE)

Solid-phase extraction is the most used sample preparation technique for the isolation, extraction, and preconcentration of parabens in environmental water samples [47,53]. Compared to the traditional liquid–liquid extraction (LLE) that poses practical problems when paraben samples are analyzed due to additional laborious steps and the high consumption of organic solvents, SPE has become the most reliable extraction technique [54,55,56]. Subsequently, a wide array of advantages such as selectivity, simplicity, higher enrichment factors, less consumption of organic solvent, shorter analysis time, no phase emulsion, higher method recovery, and more efficient isolation of target analytes from interfering compounds have been observed for SPE [57,58,59,60,61]. Due to these advantages, it has been reported that SPE is necessary to apply toward the extraction of parabens, which will ensure the separation of parabens (including separation from metabolites) and purify the sample from other co-existing molecules within the sample matrix [62,63]. This enables accuracy in terms of assessing the concentration levels of the target paraben analytes. In principle, SPE involves flowing the paraben-containing water sample through a solid adsorbent material to concentrate and isolate the parabens [64]. The adsorbed parabens on the surface of the adsorbent material are then eluted with a solvent that is capable of desorbing parabens and later analyzed using analytical techniques such as HPLC and LC–MS/MS, amongst others [63,64]. The adsorbent material and the eluent solvent are critical elements in SPE and are the determining factors of a good extraction and analysis of the paraben’s concentrations [65]. The SPE technique is usually implemented by using a small column or cartridge containing an appropriate sorbent, with octadecyl bonded silica (C18), Bond Elut, Strata-X, and Oasis HLB sorbents used [57,66]. Though most researchers resort to the commercially available Oasis HLB cartridge adsorbent, more effort has been put into newly developed adsorbent materials, as well as the optimization of eluent formulations [67,68,69].

Yengin et al. [41] prepared a metal–organic framework MIL-101 (Cr) and applied it as an adsorbent in the vortex-assisted solid-phase extraction of methyl, ethyl, propyl, and butyl parabens before HPLC–DAD analysis, demonstrating the performance of the integrated VA-SPE-HPLC method. The study evaluated the influence of analytical parameters such as pH, adsorption–desorption time, amount of adsorbent, elution solvents, desorption volume and salt effect. The optimum conditions attained were used in real water samples, and recovery values obtained with this method were in the range of 90.78–104.89% for the water matrix. The detection limits of the HPLC method for methyl, ethyl, propyl, and butyl parabens were 0.0387, 0.0322, 0.0299, and 0.0339 µg/mL, respectively. The authors concluded that the VA-SPE-HPLC procedure was effective for the extraction and determination of methyl, ethyl, propyl, and butyl parabens from water due to the vortex-induced vibrations [70]. Figure S1 demonstrates the solid phase extraction, followed by the HPLC analysis procedure for parabens extraction and quantification.

### 2.2. Rotating-Disk Sorptive Extraction (RDSE)

Rotating-disk sorptive extraction (RDSE) is an extraction/stirring integrated technique adopted as an alternative microextraction technique that uses a rotating disk coated with a sorptive material to extract/preconcentrate parabens from water samples [71,72]. RDSE is an equilibrium-based technique rather than an exhaustive microextraction technique allowing the use of laminar and powder solid phases [72,73]. In principle, the sample with the analytes of interest is added into the reaction vessel and the disk coated with the adsorbent material is rotated at a given time and temperature allowing for the extraction of parabens. Post extraction, the disk with adsorbed analytes is isolated from the aqueous matrix and a desorption solvent is used whilst rotating. The desorption solvent containing the concentrated analyte is then analyzed using analytical techniques of interest [74,75,76,77]. RDSE provides some advantages over SPE, especially by allowing for the recirculation of the sample through the extraction phase and thus maximizing its sorptive capacity [78]. In addition, the great versatility of RDSE in the study of hydrophobic and hydrophilic analytes is evidenced considering the ease of immobilization of different sorptive phases in both laminar and particulate forms [72,79]. The sorptive phase follows two configurations: (1) The sorptive phase is a polymeric film adhered to one side of the Teflon disk; (2) the Teflon disk contains a cavity loaded with a commercial sorptive phase which is later covered with a fiberglass filter sealed with a Teflon ring [79,80]. Moreover, as an alternative microextraction technique, RDSE uses low sample volumes, a configuration of the device allowing the incorporation of laminar and powder phases with different polarities (high versatility), and the lack of device deterioration during the extraction since the extracting phase is not in contact with the extraction container [81]. Various sorbents such as polydimethylsiloxane (PDMS) [78,82], nylon-6 [83], styrene-divinylbenzene (S-DVB) [84], laminar cork [85], and octadecyl silane-C18 [78], among other materials, have been used as sorptive materials for the extraction of parabens, with various polarities.

For instance, Torres et al. [86] developed and compared activated carbon extraction efficiency over common sorbents such as C18, SDVB and HLB. In this study, peanut shells, peanut shell biochar and activated carbon were assessed using rotating-disk sorptive extraction for the extraction of ethylparaben, amongst other emerging contaminants. In another study, Vieira et al. [87] investigated the viability of applying cork and montmorillonite clay modified with ionic liquid as biosorbents for the extraction of parabens such as methyl paraben, ethyl paraben, propylparaben, and isobutyl paraben using the rotating-disk sorptive extraction technique. The extraction efficiencies for the target compounds in aqueous matrices were compared to those obtained using the Octadecil Silano-C18 commercial sorbent. The proposed methods achieved limits of quantification of 0.8 μg/L for cork, 3.0 μg/L for montmorillonite clay and 6.0 μg/L for Octadecil Silano-C18. The relative recoveries from river water and tap water samples ranged from 80.3 to 118.7% and 80.0 to 119.9% for cork and montmorillonite clay modified with ionic liquid, respectively. The study showed that other adsorbents can perform better than commercial adsorbents in the extraction of parabens. Figure S2 shows the application of RDSE combined with GC–MS for the extraction and analysis of parabens.

### 2.3. Vortex-Assisted Dispersive Liquid–Liquid Extraction (VA-DLLE)

Liquid–liquid extraction is one of the oldest microextraction techniques that has evolved over the years to improve their extraction efficiency [88,89,90]. Amongst other types of liquid–liquid extraction techniques, vortex-assisted dispersive liquid–liquid extraction involves the addition of a small volume of an organic solvent to the water sample and vortexed to disperse the solvent whilst allowing for the extraction of micropollutants from the water sample to the organic solvent phase [91,92,93]. The organic solvent phase containing the extracted parabens is then separated from the water phase prior to analysis using the analytical technique of interest [93]. The main advantage of this technique is the high mass transfer between the phases, which is achieved by the ability to reach high rotational velocities without tearing the extraction device [82].

As an example, the extraction of parabens such as methylparaben, ethylparaben, propylparaben and benzylparaben in environmental water samples was developed following the optimization of selected parameters for each method of extraction. In this study [94], methods involved were cloud point extraction, vortex extraction, and liquid–liquid extraction. The optimum parameters studied included the type of solvent, solvent concentration, ratio of surfactant to water and extraction time; and there were similarities in the three extraction methods studied. The correlation coefficient for the calibration of paraben at concentration 0.2 ppm–1.0 ppm was in the range from 0.9703 to 0.9942. The limit of detection of studied paraben were 0.1627, 0.0837, 0.1156 and 0.1918 ppm, respectively. Percentage recovery for cloud point extraction, vortex extraction and liquid–liquid extraction were between 41.0–147.9%, 26.5–134.7%, and 31.4–142.4% respectively. The drawback of this study was that the solvents used were not in line with green principles.

Furthermore, an effective and fast vortex-assisted dispersive liquid–liquid extraction method was developed by Beh et al. [95], for the extraction of paraben in cosmetic samples and water samples. The paraben was determined and quantified using ultraviolet-visible (UV-Vis) spectrometry. spiked water sample extraction recoveries were in the range of 88.8–100.63%. this extraction method is fast and inexpensive for the extraction of paraben. A response surface methodology (RSM) based on the central composite design was used for the optimization of factors (composition of the extractant, volume of extractant, extraction time, centrifugation time, and centrifugation velocity) affecting the extraction efficiency of the procedure. The optimum parameters for vortex-assisted dispersive liquid–liquid extraction (VA-DLLE) is chloroform used as the extractant solvent, 5 mL volume of extractant, 3 min extraction time, 5 min centrifugation time, and 2400 rpm centrifugation velocity. The limit of detection (LOD) and the limit of quantification (LOQ) for paraben are 0.0476 and 0.1442 μg/mL, respectively. This study also did not adopt to the principles of green chemistry.

Subsequently, applying the concepts of green analytical chemistry across the whole method development is gaining more interest in using eco-friendly solvents and minimizing waste generation. Therefore, replacing conventional methods with benign alternatives has been the aim of many researchers and practitioners worldwide. Natural deep eutectic solvents (NDESs) can be considered as a green alternative to the toxic organic solvents [96]. Compared to the traditional LLE, VA-DLLME is mostly designed to follow the principles of green chemistry. This follows the development of twelve principles of green chemistry that have been designed to protect the environment and human health [97]. To this end, green solvents such as surfactants, ionic liquids, deep eutectic solvents, and bio-derived solvents have been reported as alternatives to the halogenated solvents used in traditional LLE [98].

In another study, Dalmaz et al. [99] developed a green deep eutectic solvent-based vortex-assisted liquid–liquid microextraction (DES-VALLME) procedure for the determination of parabens. The deep eutectic solvent combined DL-menthol and decanoic acid in different mole ratios and was successful in the extraction of methyl, ethyl, propyl, and butyl paraben. The study motivated that solvent selection is an extremely important issue in the liquid–liquid microextraction method, and showed that deep eutectic solvents (DES), which are in the class of green solvents, are critical for application in the VA-LLME of parabens. Figure S3 shows the application of the ultrasound-assisted liquid–liquid extraction (UA-LLE) combined with LC–MS on the extraction and analysis of heptyl parabens in highly saline water.

Online monitoring methods have been developed for detecting parabens during wastewater treatment processes [55,100,101]. The online mode presents advantages such as reusable column-type cartridges, fast elution with less solvent consumption (0.5–10 mL), minimal degradation, and no loss of analyte by evaporation resulting in reduced analysis time. The disadvantages are less material phase availability and lower analyte concentration factor. There has been an increase in the use of automated instruments that integrate extraction, purification and detection. Nascimento et al. [20] reported a method to quantify methylparaben, ethylparaben, propylparaben, and butylparaben in urban waters of São Carlos (São Paulo State, Brazil) using an online solid-phase extraction-liquid chromatography–tandem mass spectrometry (SPE-LC–MS/MS) system. The method achieved ng/L limits with good accuracy and precision (internal standards for each paraben were used). The total run cycle time took 9.5 min per sample including the extraction, clean-up and columns conditioning cycles, lower collection volumes of urban water, and solvent usage. The SPE cartridge applied showed robustness, allowing over 500 sample injections (800 µL each) with good chromatographic performance. Other studies adopting online monitoring methods were reported in China [102] and Tunisia [103].

### 2.4. Application of the Integrated Approaches for Monitoring Parabens in Water and Wastewater Matrices

**Figure 2:**
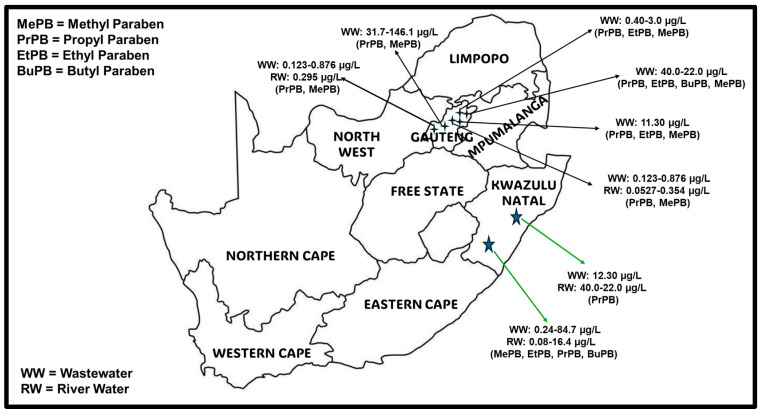
Monitoring of parabens in various river and wastewater matrices in South Africa [105,106,107,108,109,110,111,112].

The monitoring of parabens has been gaining attention due to their widespread use in personal care products and their potential environmental impact. Several studies have documented the existence of various personal care products, particularly in middle- to lower-class countries. In South Africa, as shown in Figure 2, various studies on the detection and analysis of different classes of parabens have been carried out in Gauteng and KwaZulu-Natal provinces.

Methyl and propyl parabens are most often detected in influents from wastewater at concentrations of up to 30 μg/L and 20 μg/L [104]. The latest study in South Africa [105] detected and quantified MePB (2.02–84.7 μg/L), EtPB (<0.24–24.8 μg/L), PrPB (<0.26–55.1 μg/L), and BuPB (<0.27–17.3 μg/L) in wastewater influent collected from wastewater treatment plants (WWTPs) in KwaZulu Natal (KZN) Province. Compared to other previous studies in the same region demonstrated in Figure 2 [106,107,108,109,110,111,112], the high concentrations were ascribed to the increased use of paraben-containing products and chemicals during the COVID-19 pandemic season. Interestingly, effluent wastewater from the same WWTPs showed significant reduction in parabens, with <0.19–5.43 μg/L, <0.16–5.63 μg/L, <0.17–6.89 μg/L, and <0.19–5.32 μg/L for MePB, EtPB, PrPB, and BuPB quantified, respectively. Similarly, the same study detected and quantified MePB, EtPB, PrPB, and BuPB corresponding to 2.58–123.0 μg/L, <0.24–33.6 μg/L, 3.77–73.4 μg/L, and <0.27–85.8 μg/L in influent wastewater from Gauteng Province, respectively. In effluent wastewater, concentrations ranged from 0.24–17.76 μg/L (MePB), <0.16–4.88 μg/L (EtPB), 0.69–12.5 μg/L (PrPB), and <0.19–4.73 μg/L (BuPB). This study shows that the WWTPs were able to partially reduce the concentrations of all parabens studied; however, high concentrations averaging to above 5 μg/L are still being discharged to nearby rivers and streams, highlighting the unsafe discharges of the effluents. As a result, paraben residues in the surface water of KZN and Gauteng Province were quantified by the same authors [105]. However, the concentrations in KZN (<0.08–16.4 μg/L) were higher than those in Gauteng Province (0.08–3.14 µg/L), which was inconsistent with the effluent discharges from the WWTPs. This could highlight a challenge of either non-compliance or wastewater leakages from pipes, with some of the wastewater effluent in KZN not reaching the WWTPs, but instead being discharged directly into the environment. Nonetheless, this study reported on the highest concentrations observed in South Africa, compared to other previous studies [106,107,108,109,110,111,112], as shown in Figure 2. Considering that Gauteng and KZN were reported to have increased paraben concentration during the COVID-19 pandemic, the lack of studies on the monitoring of parabens in the Western Cape water matrices, one of the economic hubs and tourist destination provinces in South Africa, particularly post the COVID-19 pandemic is concerning. Furthermore, the lack of similar studies in the Eastern Cape Province, where most of the pharmaceutical industries producing paraben-related products are located, and the province being home to the second largest population in South Africa is concerning. Other remaining provinces also lack studies on paraben residues as shown in Figure 2.

**Figure 3:**
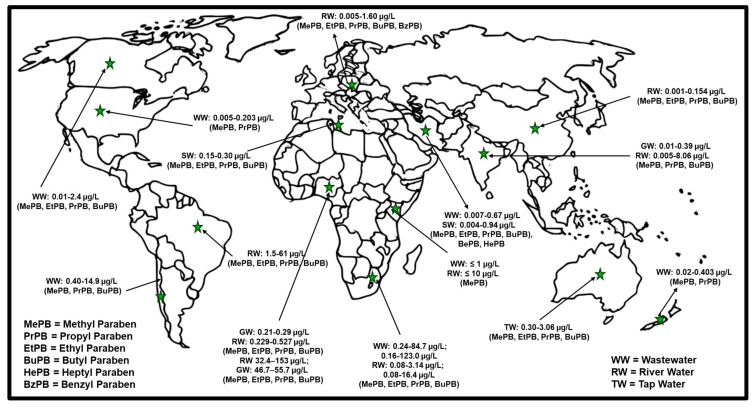
Geographic monitoring of parabens detected in surface water and wastewater worldwide [103,105,113,114,115,116,117,118,120,121,122,123,124,125,126].

Various studies on the detection and analysis of different classes of parabens have been carried out globally, as shown in Figure 3. In South America, a study carried out in Brazil [113] has shown the highest MePB concentration of 61 µg/L in the Mogi Guaçu River, with high concentrations of 26.3 µg/L, 50.5 µg/L, and 57.3 µg/L also reported for EtPB, PrPB, and BuPB, respectively. The collected samples were extracted and preconcentrated by dispersive liquid–liquid microextraction (DLLME) and later analyzed using ultra-performance liquid chromatography coupled with electrospray ionization in tandem with mass spectrometry (UPLC-ESI-MS/MS). The high concentrations were ascribed to the low removal efficiency of chemicals in WWTPs leading to the discharge of both treated and untreated wastewater that could be the source of parabens in the Mogi Guaçu River. The authors suggested frequent and integrated monitoring in rivers and wastewater treatment plants, especially as there are no clear actions in their legislation to deal with paraben issues. The concentrations detected in wastewater in Chile [114] for MePB, PrPB, and BuPB after extraction using rotating-disk sorptive extraction (RDSE) technology with ultra-high performance liquid chromatography/electrospray ionization source (ESI) and time of flight mass spectrometry (UHPLC-ESI-TOF MS) were in the range of 0.40–14.9 µg/L. In Asia, a study carried out in India [115] has shown concentrations as high as 8 µg/L in drinking water. The high concentrations were attributed to the production, consumption, and discharge status in the area as many pharmaceutical industries have relocated from the West to the East. Studying the concentrations of the parabens in wastewater influent and effluent might give a bigger picture of the high concentrations detected. The concentrations in China (0.001–0.154 µg/L) [116] and Iran (0.004–0.94 µg/L) [117] in river and seawater, respectively, were found to be lower than those detected in India for the same analytes. In Africa, MePB was detected in Kenyan wastewater and river water at 1 µg/L [118] and 10 µg/L [119], respectively, after extraction using SPE and detected using UHPLC. The high concentrations detected in river water were in the urban area and ascribed to the increased use of cosmetics. As described previously, the high MePB, EtPB, PrPB, and BuPB were extracted using an offline solid-phase extraction C18 SPE cartridge-es and detected with HPLC–DAD in the range of 0.24–84.7 µg/L and 0.16–123.0 µg/L in wastewater; 0.08–3.14 µg/L and 0.08–16.4 µg/L in river water were ascribed to the increased use of paraben products during the COVID-19 pandemic [105]. Similar analytes were studied in a recent study (2024) in Nigeria, and the average concentrations were 32.4–153 µg/L, 53.4–80.1 µg/L, and 83.2–132 µg/L for surface water and 46.7–55.7 µg/L, 53–117 µg/L, and 62.4–118 µg/L for groundwater in Osun, Oyo, and Lagos States, respectively, after extraction using SPE/UHPLC/MS [120]. Expectedly, MePB was the most frequently detected paraben with average concentrations of 153 µg/L and 117 µg/L in surface water and groundwater, respectively. The study was conducted amid the pandemic and the increased use of paraben products could have escalated the concentrations. The results were compared with another study in Nigeria carried out before COVID-19, with similar analytes analyzed, but the concentrations only ranged from 0.1–0.5 µg/L in ground and river water after SPE/HPLC-UV extraction and analysis [6]. These results confirm the impact COVID-19 had on the increased use of parabens, and many countries worldwide could have detrimental concentrations in their water matrices unless the monitoring of parabens is carried out and mitigated. Tunisia also reported low concentrations in the range of 0.015–0.30 before COVID-19 in 2022 [103]. Other studies carried out for MePB, PrPB, and BuPB analysis in Vietnam [121], Poland [122], USA [27,123,124], Canada [30], Australia [125], and New Zealand [126] detected concentrations below 3.1 µg/L as shown in Figure 3; however, all these concentrations were detected before the COVID-19 pandemic.

Worryingly, many countries such as Indonesia, Pakistan, Bangladesh, Russia, Ethiopia, Mexico, Japan, Egypt, Philippines, Congo, Iran, Turkey, Germany, and Thailand sit in the top 20 list of the most populated countries in the world and have shown a lack of recent studies on monitoring parabens in water matrices. Considering the diverse range of age groups in their populations, a wide range of paraben-containing products are being used, thus it would be beneficial for them to analyze the concentrations of various classes of parabens, particularly post-COVID-19 waves. As parabens are used as preservatives in food items and cosmetics, there is an increased risk in contracting diseases such as diabetes and even obesity, especially in countries with aging populations and those highly dependent on processed foods. The results from the studies analyzed indicate that the risk associated with paraben pollution may be higher in South Africa, Brazil, and Nigeria. However, due to the known risk of parabens exerting endocrine-disrupting effects, more monitoring of these compounds in surface waters needs to be done, especially since recent monitoring studies in most countries worldwide are lacking, particularly post the COVID-19 pandemic. There are limited reports on the occurrence of parabens in various water matrices, especially in the African and Asian continents.

## 3. Treatment Techniques for Parabens

One of the key strategies in the water resource strategy for achieving the delicate balance between water supply and demand is wastewater reclamation [127]. The removal efficiency of parabens in selected Gauteng WWTPs during the third wave of COVID-19 was reported by [105]. The results showed that high removal efficiencies of 77.3–100% were recorded, implying that the levels of parabens discovered in influent wastewater samples were large or eliminated in most cases. Despite reaching 100% removal efficiency in some WWTPs, substantial amounts of parabens (up to 15.5 μg/L) were still found in effluent samples. These findings demonstrated that traditional WWTPs are not designed to completely remove emerging pollutants. Furthermore, the chemical stability and nonbiodegradability of parabens may account for their inadequate removal by standard WWTPs [128].

### 3.1. Physical Adsorption Processes

Parabens are removed from water using physical adsorption processes that capture these compounds on the surface of adsorbent materials via weak van der Waals forces. Zeolite [129], activated carbon [130,131], and biochar [132] are commonly used as adsorbents because of their high surface area and porosity, which improve paraben adsorption. The hydrophobic nature of parabens aids in their attraction to the adsorbent non-polar regions.

#### 3.1.1. Diverse Adsorbents for Paraben Adsorption in Water: Applications

The efficiency of paraben removal is determined by the adsorbent’s pore size, surface chemistry, and operating conditions such as temperature and pH. Physical adsorption is regarded as an environmentally friendly and efficient method for reducing paraben contamination in water.

Ref. [133] investigated the use of recyclable magnetic waste tire-activated carbon-chitosan composite as an effective adsorbent for rapid and simultaneous removal of methylparaben and propylparaben from aqueous solution and wastewater. The initial concentrations of methylparaben (MP) and propylparaben (PP) in influent wastewater were determined by solid-phase extraction using the prepared adsorbent, and verification of the results was done using a commercial SPE cartridge (Table 1). The removal efficiency obtained was 100% for both methylparaben and propylparaben. The results show that magnetic adsorbent has benefits including convenient usage, affordability, and the ability to be easily removed from liquid solutions using an external magnetic field. Table 2 shows experimental conditions and adsorption parameters for the removal of parabens using various adsorbents. Overall, Zeolitic imidazolate-67 modified by Fe_3_O_4_ nanoparticles and Biochar–CoFe_2_O_4_ nanocomposite exhibit better adsorption capacities than the other materials, demonstrating their efficacy in paraben removal under the tested conditions (Table 2).

Insights into paraben adsorption by metal−organic frameworks for analytical applications were investigated [134]. The computational analysis found that PPB adsorption in the two pore types of the CIM-81 framework is energy-efficient, with a maximum loading capacity of 291.9 mg g^−1^. The adsorption isotherm yielded a value of 283 mg g^−1^. These values are higher than any previously reported values for PPB adsorption in the literature. MOFs were successfully used in analytical techniques to determine a series of CECs in water.

Pourmohammad et al. [135] investigated the removal of benzyl paraben from wastewater using Zeolitic Imidazolate-67 modified by Fe_3_O_4_ nanoparticles and response surface methodology. Results show that Zeolitic Imidazolate-67, modified with Fe_3_O_4_ nanoparticles was effective in removing benzyl paraben from wastewater through adsorption utilizing the response surface technique. Furthermore, it can serve as a reusable adsorbent, providing an economically viable option for industrial wastewater treatment and high-quality efficiency.

**Table 1:** Initial concentration in µg L^−1^ of methylparaben (MP) and propylparaben (PP) from influent water samples. Reproduced with permission from [133].

Samples	MP Initial Concentration	PP Initial Concentration
Influent 1	0.947 ± 3	0.108 ± 1
Influent 1	0.889 ± 13	1.988 ± 9
Influent 1	1.293 ± 20	2.113 ± 15

**Table 2:** Experimental conditions and adsorption parameters for the removal of parabens using various adsorbents.

Adsorbent Material	Parabens	Initial Concentration (mg/L)	pH	Adsorption Capacity (mg/g)	Ref
Modified cellulose	Methylparaben Butylparaben	15 25	4	9.58 12.03	[136]
Carboxymethylcellulose/Hydrotalcite Bionanocomposites	4-methyl paraben	25	7	4	[137]
Zeolitic imidazolate-67 modified by Fe_3_O_4_ nanoparticles	Propylparaben	10	6	117.5	[138]
Activated Carbon and Activated Olive Stones	Methylparaben	50	3	20 90	[139]
Activated Carbon	Methylparaben Ethylparaben	20	8	150 160	[130]
Magnetic nanoparticles PS/Fe_3_O_4_	Methylparaben Ethylparaben	5	7	0.60 3.29 3.54	[140]
TiO_2_ nanoparticle-loaded activated carbon	Propyl parabene	12	4	120	[141]

#### 3.1.2. Optimizing Water Treatment: Engineering Feasibility, Cost-Effectiveness, and Comprehensive Solutions

Physical adsorption is a widely used approach for removing parabens in water matrices, primarily employing adsorbents such as activated carbon, biochar, and synthetic resins. This method is technically feasible and integrates well into existing water treatment systems. Due to its scalability, adsorption systems can be applied effectively in both small and large-scale facilities [142]. The operational simplicity associated with adsorption, requiring minimal technical expertise, makes it suitable for deployment in areas with limited resources [143]. However, the saturation of adsorbents over time necessitates periodic regeneration or disposal, which may limit continuous operations and efficiency, particularly if different types of parabens require specific adsorbents [144]. The initial capital costs of physical adsorption treatments are moderate, particularly when using readily available materials like activated carbon. However, significant ongoing operational costs arise from the need for adsorbent replacement or regeneration. While activated carbon can be regenerated, enabling reuse across multiple cycles, this process does involve additional energy costs, which can impact economic feasibility, especially for larger-scale applications. Notably, ref. [131] has shown that activated carbon can be regenerated up to 15 times with high removal efficiencies of 91.37% for methylparaben (MP) and 87.13% for propylparaben (PP), making it an effective option for sustained use in certain contexts. However, even with high removal rates, each regeneration cycle generates saturated adsorbents containing potentially hazardous contaminants, which require careful handling and disposal, adding further to waste management costs [145].

To improve cost-effectiveness and efficiency in water treatment, combining adsorption with advanced oxidation processes (AOPs) or biological treatments has shown significant promise [146].

Future research and development are likely to focus on integrating multiple technologies for the effective removal of antibiotics from water. This approach aims to enhance treatment efficiency and sustainability. Additionally, advancements in innovative materials and alternative treatment methods—such as ionizing radiation and constructed wetlands—are expected to remain at the forefront of research [147]. In these hybrid treatment systems, adsorption can serve as a pre-treatment step, effectively reducing contaminant levels before secondary processes. This strategy optimizes resource usage and may significantly reduce costs. To make adsorption even more feasible for large-scale applications, ongoing research is exploring low-cost adsorbents. Examples include materials derived from agricultural waste or modified natural sources, which offer economical and effective solutions for contaminant removal [148].

### 3.2. Overview of Degradation of Parabens

The degradation of parabens is primarily achieved through advanced oxidation processes (AOPs) like ozonation, photocatalysis, and Fenton reactions, which effectively break down paraben molecules into less harmful byproducts [149]. These processes can be highly effective but often require specific conditions, such as UV light sources for photocatalysis or chemical reagents for Fenton reactions, which can limit scalability in areas without access to advanced infrastructure [150]. Additionally, degradation systems demand close monitoring of factors like pH, temperature, and contaminant concentration, which can add to the technical complexity.

#### 3.2.1. Paraben Removal Techniques: Comparative Methods and Practical Applications

Table 3 shows that catalytic ozonation and UV/H_2_O_2_ processes are the most efficient in degrading parabens. Photocatalytic approaches are promising but may need further optimization, especially in catalyst design. The effectiveness of these methods is influenced by variables such as light source, pH, and reaction time, which must be fine-tuned based on the specific application. Some methods, such as UV/H_2_O_2_, are pH-dependent, with different parabens degrading efficiently at different pH conditions (e.g., methylparaben at pH 9–10 and dihalo-parabens at pH 4–6). This implies that treatment conditions must be carefully monitored for the best results.

Gmurek et al. [151] studied the photodegradation of single and mixture parabens—kinetic, identification byproducts, and cost-efficiency analysis. The most reactive chemicals among the contaminants examined were pHBA and BeP. The addition of hydrogen peroxide significantly accelerated the degradation of pollutants, resulting in the complete depletion of the blend in 30 min compared to 8 h with only UVC. The most reactive chemicals among the contaminants examined were pHBA and BeP.

**Table 3:** Methods for degradation and removal of parabens.

Adsorbent Material	Parabens	Initial Concentration (mg/L)	pH	Adsorption Capacity (mg/g)	Ref
Photo catalysis	Benzyl-paraben	Graphitic carbon nitride (g-C_3_N_4_) and monoclinic bismuth vanadate (BiVO_4_), was fabricated by a facile hydrothermal synthesis and employed to treat benzyl-paraben (BzP).	55	Visible light irradiation for 150 min when using 5% GCN/BiVO_4_ as catalyst.	[152]
Catalytic Ozonation	Methyl-, ethyl- and propyl-paraben	The g-C_3_N_4_ was prepared following a thermal polymerization method. Melamine (MCN), urea (UCN), and thiourea (TCN), used as precursors.	92	The catalyst load used in all reactions was 200 mg L^−1^. The concentration of each paraben in all reactions was 1 mg L^−1^.	[153]
Photocatalytic degradation	Methyl-, ethyl- and propyl-paraben	Thermally exfoliated carbon nitride (GCN-500).		Under narrow visible light irradiation (kmax = 417- nm) provided by a LED source.	[154]
Photocatalytic	Methylparaben	EB/Ag_3_PO_4_/AgBr (30%) hybrid was successfully synthesized using a deposition–precipitation and in situ anion-exchange methods.	91		[155]
Degradation of parabens by Graphene family nanocomposite (GFNs) in the presence of H_2_O_2_	Methylparaben	Generation of ∙OH from H_2_O_2_ as triggered by (GFN). ∙OH trapping agent, was added into the GFNs-H_2_O_2_ system.	48	[MG]_0_ = 400 mg/L, [RGO]_0_ = 200 mg/L, [MeP]_0_ = 1–80 mg/L, [H_2_O_2_]_0_ = 100 mM, [HA]_0_ = 10 mg/L, pH = 6.0, and reaction time of 96 h.	[156]
UV/H_2_O_2_ process	Methlparaben dihalo-parabens	The HO∙-initiated reaction mechanisms of MeP and halo-parabens were calculated, which included radical adduct formation (RAF), hydrogen atom abstraction (HAA), and single electron transfer (SET).	>90 100	In 10 min, dihalo-parabens appeared at pH = 4–6, while MeP appeared at pH = 9–10. UV/H_2_O_2_ treatment with a H_2_O_2_ concentration of 10 mg L^−1^ and exposure to a UV fluence of 2000 mJ cm^−2^.	[157]

#### 3.2.2. Enhancing Water Treatment: Engineering Viability, Economic Efficiency, and All-Encompassing Solutions

The costs associated with degradation methods include high initial investments in equipment and infrastructure. Systems such as ozonation require substantial initial investments, and operational costs can be driven up by the consumption of reagents, as seen in Fenton reactions where the ongoing addition of hydrogen peroxide is necessary [158]. Unfortunately, some parallel reactions take place, which means that the hydroxyl radicals are not only used to break down organic matter but also to create other species or radicals with lower oxidative power (the scavenging effect of HO•). Furthermore, this results in the undesirable consumption of H_2_O_2_ [158]. Maintenance costs in UV-based systems are high, primarily due to the regular replacement of components required to sustain operational effectiveness. This increases the overall expenses, particularly on a large scale, as the energy and resource demands may challenge economic feasibility. A balanced approach to address these costs is to incorporate adsorption as a pre-treatment method to reduce contaminant concentration before applying advanced oxidation processes (AOPs). This approach can lower the reagent and energy demands in the subsequent degradation stage [159]. In one study, ref. [159] investigated a combined O_3_/UV oxidation and activated carbon adsorption system for removing emerging contaminants (ECs) from hospital wastewater. This hybrid system, combining ozone/UV with granular activated carbon (GAC), was effective in removing contaminants like pharmaceuticals and pesticides, enhancing economic viability by reducing energy and reagent needs. Further, integrating byproduct recovery processes, such as waste-to-energy initiatives, can help offset operational costs by reusing byproducts in secondary applications. Bioconversion plays a crucial role in meeting the rising demand for raw materials, controlling production costs, minimizing environmental impact, and managing waste [160]. The shift toward waste management now emphasizes the circular economy concept, where resources, energy, and materials are continuously reused and recycled [161]. Developing a CE strategy will help to combat the global crisis of climate change, biodiversity loss, and pollution.

### 3.3. Membranes

Membrane separation technologies are widely used for seawater desalination [162], water supply plant operations [163], and sewage water treatment [164] due to their low chemical dosage, easy maintenance, and small footprint. Microfiltration (MF), ultrafiltration (UF), nanofiltration (NF), and reverse osmosis (RO) membranes are examples of conventional membranes. Membrane filtration has several advantages, including controlling mass transport and fouling, separating solid–liquid particles, and removing organic matter and inorganic contaminants [165].

#### 3.3.1. Emerging Membrane-Based Solutions for Effective Paraben Removal from Wastewater

López-Ortiz et al. [166] studied the use of combined treatments for reducing parabens in surface waters: ion-exchange resin and nanofiltration [166]. The selected parabens included methylparaben, ethylparaben, propylparaben, and butylparaben. MIEX^®^ DOC and MIEX GOLD resins, as well as NF-90 and DESAL-HL nanofiltration membranes, were used in this study. Results show that the NF-90 membrane resulted in an improvement for propylparaben and butylparaben levels. Combining DOC resin with the DESAL-HL membrane resulted in the highest removal efficiency. This study is limited as there is not much reported on the treatment of parabens using ion exchange and nanofiltration. Bhattacharta et al. [167] investigated an indigenously developed CuO/TiO_2_ coated ceramic ultrafiltration (UF) membrane for the removal of emerging contaminants such as parabens. A CuO/TiO_2_ nanoparticle-based ceramic ultrafiltration membrane removed phthalates and parabens from synthetic systems at concentrations ranging from 10 to 1000 ppb with over 99% efficiency after 60 min of filtration at 5 bar pressure. Membrane-based treatment of persistent pollutants can be scaled up for industrial and domestic applications.

Kohli and co-authors [168] attempted to remove ethylparaben from aqueous solutions using pseudo-emulsion and hollow fiber membranes. The study examined how feed concentration, carrier, and stripping phase affect EP removal success. The pseudo-emulsion hollow fiber strip dispersion (PEHFSD) system successfully removed 100% of EP from the aqueous feed phase under optimal conditions. The overall permeability coefficient for the PEHFSD system was 3.55 × 10^−6^ m/s. The mass transfer coefficients for the aqueous and membrane phases were estimated at 8.35 × 10^−6^ m/s and 5.16 × 10^−6^ m/s, respectively. The study found that membrane module and pseudo-emulsion characteristics, in addition to operational factors, significantly impact EP removal.

#### 3.3.2. Economic Effectiveness and Engineering Feasibility Linked to Membrane Technology

Membrane filtration, including nanofiltration (NF) and reverse osmosis (RO), is highly effective for paraben removal, operating through selective filtration that separates contaminants based on size and chemical properties [169]. Membrane filtration systems are highly versatile, capable of scaling to meet the needs of both small- and large-scale applications. However, membrane fouling—particularly from organic compounds such as parabens—poses a persistent challenge. Fouling can significantly reduce system efficiency, requiring frequent cleaning or even replacement of membranes to maintain consistent performance. This degradation in membrane performance often demands extensive replacement of filtration components, especially in large-scale operations, where the high cost of membrane materials and infrastructure limits broader adoption. According to Osman et al. [170], the high capital costs associated with membrane filtration systems restrict their wide-scale application in water treatment, and the design of high-performance, cost-effective membrane materials at a large scale remain a challenging task.

To address these issues, Diez [171] proposed several strategies to mitigate membrane fouling and biofouling. One effective approach they identified involved adding polyvinylpyrrolidone (PVP) to the casting solution of polyethersulfone (PES) ultrafiltration (UF) membranes, which successfully modified pore size to reduce fouling. More recent strategies are built on this foundation by employing advanced techniques like grafting, coating, and blending to further improve membrane performance and fouling resistance [172]. The economic and operational feasibility of membrane systems is also heavily influenced by energy costs, especially in pressure-driven configurations like reverse osmosis (RO), which require high pressures to maintain filtration effectiveness. This pressure requirement not only raises energy consumption but also drives up operational costs, particularly when fouling increases the resistance to flow. Therefore, reducing fouling is integral to lowering these costs, supporting the engineering and financial feasibility of RO and similar systems.

Ref. [173] has explored innovative approaches to address this energy issue, including the use of a saturated CO_2_ solution to remove sodium alginate fouling from RO membranes. By injecting varying concentrations of Ca^2^⁺ ions to accelerate fouling, and testing different operating conditions, Alnajjar et al. [173] found that this method could reduce both the chemical disposal costs and environmental impacts associated with conventional cleaning methods. Additionally, minimizing fouling lowers the pumping costs and energy demands of RO systems, while improving the membrane recovery ratio, further reducing operational costs. Moreover, exploring energy-efficient innovations, such as forward osmosis or electrically assisted filtration, may help reduce operational costs and make membrane filtration more viable for widespread application [174].

## 4. Challenges Associated with Parabens Treatment Along with Removal Mechanisms

The removal of paraben preservatives commonly used in personal care products presents various challenges due to their persistence in the environment, potential toxicity, and the difficulty in achieving complete degradation. To mitigate these risks and protect both human health and ecosystems, it is essential to develop effective treatment methods [175,176]. Approaches like advanced oxidation processes, biodegradation, and filtration play a vital role in minimizing environmental impacts and supporting sustainable water management. Table 4 summarizes the main challenges associated with paraben removal and highlights the mechanisms involved in addressing them.

## 5. Conclusions and Future Perspectives

Parabens are emerging contaminants of concern in water systems worldwide. The review highlights the need for improved monitoring techniques, particularly for low-concentration detection, and the necessity of advancing treatment methods to effectively remove parabens without generating harmful byproducts. Solid-phase extraction has been explored as the most effective sample preparation technique for parabens extraction and preconcentration in a water matrix [186,187]. In most studies, the use of the commercial OSB adsorbent has been reported; however, recent studies have started exploring the use of other adsorbents such as magnetite, carbon nanotubes, chitosan, amongst others that can interact with parabens with high extraction efficiencies. However, studies around the exploration of these adsorbents still need further studies to understand the adsorption processes and improve the extraction efficiencies. The quantification of parabens using analytical techniques such as HPLC, LC–MS and GC–MS are mostly dependent on the extraction and preconcentration methods to ensure effective detection and quantification with low detection and quantification limits. This highlights the importance of sample preparation of parabens to extract, isolate, and preconcentrate using developed adsorbents. Recent global trends have shown the importance of sample preparation in attaining high extraction efficiencies and quantifying concentrations with analytical techniques. It is noteworthy that the selectivity and sensitivity of the analytical techniques for the quantification of parabens have a direct correlation with the sample preparation method. While promising methods such as AOPs treatments show potential, challenges remain in terms of cost, scalability, and environmental impacts. The lack of research on ion-exchange and nanofiltration treatment techniques for treating parabens creates a critical gap. Addressing this through targeted investigations could broaden the range of technologies available for reducing paraben levels in drinking water and wastewater systems. Advancing knowledge in this area will not only aid in the treatment of parabens but may also contribute to the removal of other emerging contaminants with similar chemical properties. The regulation of parabens has been observed to vary by region, with specific guidelines to ensure consumer safety outlined from different regions worldwide. For instance, the European Union restricts the concentration of parabens in cosmetics to a maximum concentration of 0.4–0.8%, and similar measures are applied worldwide through different agencies, depending on the country (e.g., FDA in the USA) (Table S1). Forbidden and allowed compounds in the PCPs’ formulation also vary a lot among countries. For instance, certain parabens, such as isopropylparaben, isobutylparaben, phenylparaben, benzylparaben, and pentylparaben, are banned due to safety concerns in South Africa. Furthermore, 27 compounds are allowed in Europe, while only 16 and 32 are permitted in the USA and Africa, respectively. Even the allowed proportion of each compound varies amongst regions. Continued research and the development of more efficient, sustainable water treatment technologies are essential to mitigating the risks posed by parabens. Additionally, global regulatory standards need to be strengthened to ensure the safety of water resources.

## Figures and Tables

**Table 4:** Challenges and mechanisms in the removal of parabens from water systems.

Challenge	Description	Removal Mechanisms	References
Chemical Stability	Parabens exhibit stability in various environmental conditions, making them resistant to conventional treatment methods.	Advanced oxidation processes (AOPs) such as UV/O_3_, photocatalysis (e.g., TiO_2_/UV), and Fenton’s reagent can break down paraben structures.	[157,177]
Presence of co-contaminants	Parabens frequently coexist with other organic and inorganic compounds, which can disrupt treatment processes and reduce removal efficacy.	Adsorption with activated carbon or biochar can selectively remove parabens and other contaminants. Pre-treatment can also increase selectivity.	[178]
High water solubility	Due to their high solubility, parabens remain dissolved in water and are difficult to remove using traditional filtration.	Adsorption onto activated carbon or resins, and membrane filtration techniques such as nanofiltration (NF) or reverse osmosis (RO).	[179,180]
Formation of toxic byproducts	Some treatment methods can lead to the formation of toxic byproducts that may persist or be harmful to the environment.	Use of biodegradable adsorbents or bioremediation with microbes that metabolize parabens with minimal byproduct formation.	[181,182]
Variability in paraben structure	Parabens come in different forms (e.g., methylparaben, ethylparaben), each with unique chemical properties, which can affect removal efficiency.	Sequential or hybrid treatment methods, combining techniques such as adsorption followed by AOPs, to target various paraben types.	[183,184]
Cost of treatment methods	Advanced treatment methods such as AOPs and specialized adsorbents can be costly, particularly for large-scale applications.	Combined treatment systems (e.g., adsorption pre-treatment followed by oxidation) to reduce overall operational costs.	[185]

## Data Availability

No new data was generated, and all data included in the article is referenced.

## Supplementary Materials

The following supporting information can be downloaded at: https://www.mdpi.com/article/10.3390/molecules29235533/s1. Figure S1: Fe_3_O_4_@rGO-DSPE method was combined with HPLC-UV for trace paraben determination. Reproduced with permission from [188]; Figure S2: Extraction of parabens for their determination in water samples by rotating-disk sorptive extraction. Reproduced with permission from [74]. Figure S3: Sensitive and accurate ultrasound-assisted liquid-liquid extraction (UA-LLE) method for heptyl paraben extraction in saline water. Reproduced with permission from [189]. Table S1: Policy regulations of parabens in various countries [190,191,192,193,194,195].

## References

[1] Dey S., Bano F., Malik A. (2019). Pharmaceuticals and Personal Care Product (PPCP) Contamination—A Global Discharge Inventory. Pharmaceuticals and Personal Care Products: Waste Management and Treatment Technology Emerging Contaminants and Micro Pollutants.

[2] Archer E., Holton E., Fidal J., Kasprzyk-Hordern B., Carstens A., Brocker L., Kjeldsen T.R., Wolfaardt G.M. (2023). Occurrence of Contaminants of Emerging Concern in the Eerste River, South Africa: Towards the Optimisation of an Urban Water Profiling Approach for Public- and Ecological Health Risk Characterisation. Sci. Total Environ..

[3] Giri S. (2021). Water Quality Prospective in Twenty First Century: Status of Water Quality in Major River Basins, Contemporary Strategies and Impediments: A Review. Environ. Pollut..

[4] Petric Z., Ruzić J., Zuntar I. (2021). The Controversies of Parabens—An Overview Nowadays. Acta Pharm..

[5] Błedzka D., Gromadzińska J., Wasowicz W. (2014). Parabens. From Environmental Studies to Human Health. Environ. Int..

[6] Bolujoko N.B., Ogunlaja O.O., Alfred M.O., Okewole D.M., Ogunlaja A., Olukanni O.D., Msagati T.A.M., Unuabonah E.I. (2022). Occurrence and Human Exposure Assessment of Parabens in Water Sources in Osun State, Nigeria. Sci. Total Environ..

[7] Fransway A.F., Fransway P.J., Belsito D.V., Yiannias J.A. (2019). Paraben Toxicology. Dermatitis.

[8] Pan J., Liu P., Yu X., Zhang Z., Liu J. (2023). The Adverse Role of Endocrine Disrupting Chemicals in the Reproductive System. Front. Endocrinol..

[9] Hager E., Chen J., Zhao L. (2022). Minireview: Parabens Exposure and Breast Cancer. Int. J. Environ. Res. Public Health.

[10] Dasmahapatra A.K., Chatterjee J., Tchounwou P.B. (2024). A Systematic Review of the Toxic Potential of Parabens in Fish. Front. Toxicol..

[11] Chakraborty A., Adhikary S., Bhattacharya S., Dutta S., Chatterjee S., Banerjee D., Ganguly A., Rajak P. (2023). Pharmaceuticals and Personal Care Products as Emerging Environmental Contaminants: Prevalence, Toxicity, and Remedial Approaches. ACS Chem. Health Saf..

[12] Rehman M.U., Nisar B., Mohd Yatoo A., Sehar N., Tomar R., Tariq L., Ali S., Ali A., Mudasir Rashid S., Bilal Ahmad S. (2024). After Effects of Pharmaceuticals and Personal Care Products (PPCPs) on the Biosphere and Their Counteractive Ways. Sep. Purif. Technol..

[13] Lincho J., Gomes J., Martins R.C. (2021). Paraben Compounds—Part II: An Overview of Advanced Oxidation Processes for Their Degradation. Appl. Sci..

[14] Hasse Palharim P., Lastre-Acosta A.M., Mierzwa J.C., Teixeira A.C.S.C. (2022). Influence of Low and High Dosages of Methyl and Propyl Parabens on Membrane Bioreactor (MBR) Performance. Sep. Sci. Technol..

[15] Maia C., Sousa C.A., Sousa H., Vale F., Simões M. (2023). Parabens Removal from Wastewaters by Microalgae—Ecotoxicity, Metabolism and Pathways. Chem. Eng. J..

[16] Lee W., Marcotullio S., Yeom H., Son H., Kim T.H., Lee Y. (2022). Reaction Kinetics and Degradation Efficiency of Halogenated Methylparabens during Ozonation and UV/H_2_O_2_ Treatment of Drinking Water and Wastewater Effluent. J. Hazard. Mater..

[17] Eggen T., Vogelsang C. (2015). Occurrence and Fate of Pharmaceuticals and Personal Care Products in Wastewater. Comprehensive Analytical Chemistry.

[18] Dhaka S., Kumar R., Lee S.H., Kurade M.B., Jeon B.H. (2018). Degradation of Ethyl Paraben in Aqueous Medium Using Advanced Oxidation Processes: Efficiency Evaluation of UV-C Supported Oxidants. J. Clean. Prod..

[19] Shyamalagowri S., Shanthi N., Manjunathan J., Kamaraj M., Manikandan A., Aravind J. (2023). Techniques for the Detection and Quantification of Emerging Contaminants. Phys. Sci. Rev..

[20] Nascimento S., Cass Q. (2021). An Online Solid Phase Extraction-Liquid Chromatography Tandem Mass Spectrometry Method for the Analysis of Parabens in Urban Waters. J. Braz. Chem. Soc..

[21] Pastor-Belda M., Marín-Soler L., Campillo N., Viñas P., Hernández-Córdoba M. (2018). Magnetic Carbon Nanotube Composite for the Preconcentration of Parabens from Water and Urine Samples Using Dispersive Solid Phase Extraction. J. Chromatogr. A.

[22] Chen P., Wang J., Xue Y., Wang C., Sun W., Yu J., Guo H. (2024). From Challenge to Opportunity: Revolutionizing the Monitoring of Emerging Contaminants in Water with Advanced Sensors. Water Res..

[23] Werner J., Grześkowiak T., Zgoła-Grześkowiak A. (2022). A Polydimethylsiloxane/Deep Eutectic Solvent Sol-Gel Thin Film Sorbent and Its Application to Solid-Phase Microextraction of Parabens. Anal. Chim. Acta.

[24] Pena-Pereira F., Bendicho C., Pavlović D.M., Martín-Esteban A., Díaz-Álvarez M., Pan Y., Cooper J., Yang Z., Safarik I., Pospiskova K. (2021). Miniaturized Analytical Methods for Determination of Environmental Contaminants of Emerging Concern—A Review. Anal. Chim. Acta.

[25] Moscoso-Ruiz I., Navalón A., Rivas A., Zafra-Gómez A. (2023). Presence of Parabens in Children’s Faeces. Optimization and Validation of a New Analytical Method Based on the Use of Ultrasound-Assisted Extraction and Liquid Chromatography-Tandem Mass Spectrometry. J. Pharm. Biomed. Anal..

[26] Gao X., Xu K., Chi M., Li J., Suo L., Zhu L., Chen K., Mu J. (2021). Determination of Four Parabens in Cosmetics by High-Performance Liquid Chromatography with Magnetic Solid-Phase and Ionic Dispersive Liquid-Liquid Extraction. Rev. Anal. Chem..

[27] Wei F., Mortimer M., Cheng H., Sang N., Guo L.-H. (2021). Parabens as Chemicals of Emerging Concern in the Environment and Humans: A Review. Sci. Total Environ..

[28] Derisso C.R., Pompei C.M.E., Spadoto M., da Silva Pinto T., Vieira E.M. (2020). Occurrence of Parabens in Surface Water, Wastewater Treatment Plant in Southeast of Brazil and Assessment of Their Environmental Risk. Water Air Soil. Pollut..

[29] Pereira A.R., Simões M., Gomes I.B. (2023). Parabens as Environmental Contaminants of Aquatic Systems Affecting Water Quality and Microbial Dynamics. Sci. Total Environ..

[30] Lincho J., Martins R.C., Gomes J. (2021). Paraben Compounds—Part I: An Overview of Their Characteristics, Detection, and Impacts. Appl. Sci..

[31] Vale F., Sousa C.A., Sousa H., Santos L., Simões M. (2022). Parabens as Emerging Contaminants: Environmental Persistence, Current Practices and Treatment Processes. J. Clean. Prod..

[32] Wei F., Cheng H., Sang N. (2022). Comprehensive Assessment of Estrogenic Activities of Parabens by in Silico Approach and in Vitro Assays. Sci. Total Environ..

[33] Ślósarczyk K., Jakóbczyk-Karpierz S., Różkowski J., Witkowski A.J. (2021). Occurrence of Pharmaceuticals and Personal Care Products in the Water Environment of Poland: A Review. Water.

[34] Sousa H., Sousa C.A., Vale F., Santos L., Simões M. (2023). Removal of parabens from wastewater by Chlorella vulgaris-bacteria co-cultures. Sci. Total Environ..

[35] Martín-Pozo L., de Alarcón-Gómez B., Rodríguez-Gómez R., García-Córcoles M.T., Çipa M., Zafra-Gómez A. (2019). Analytical Methods for the Determination of Emerging Contaminants in Sewage Sludge Samples. A Review. Talanta.

[36] Kanu A.B. (2021). Recent Developments in Sample Preparation Techniques Combined with High-Performance Liquid Chromatography: A Critical Review. J. Chromatogr. A.

[37] Alahmad W., Kaya S.I., Cetinkaya A., Varanusupakul P., Ozkan S.A. (2023). Green Chemistry Methods for Food Analysis: Overview of Sample Preparation and Determination. Adv. Sample Prep..

[38] Lou Y., Xu Q., Chen J., Yang S., Zhu Z., Chen D. (2023). Advancements in Sample Preparation Methods for the Chromatographic and Mass Spectrometric Determination of Zearalenone and Its Metabolites in Food: An Overview. Foods.

[39] More D., Khan N., Tekade R.K., Sengupta P. (2024). An Update on Current Trend in Sample Preparation Automation in Bioanalysis: Strategies, Challenges and Future Direction. Crit. Rev. Anal. Chem..

[40] Hajeb P., Zhu L., Bossi R., Vorkamp K. (2022). Sample Preparation Techniques for Suspect and Non-Target Screening of Emerging Contaminants. Chemosphere.

[41] Yengin C., Gumus Z.P., Ilktac R., Elci A., Soylak M. (2023). Vortex-Assisted Solid Phase Extraction on MIL-101 (Cr) of Parabens in Waters and Cosmetics by HPLC–DAD. J. Iran. Chem. Soc..

[42] Nasrollahi S.S., Moosavi N.S., Yamini Y. (2023). Determination of Parabens in Different Samples Using Green Analytical Chemistry Approaches since 2015. TrAC Trends Anal. Chem..

[43] YILMAZ P.K., Kolak U. (2023). Ultrasound-and Vortex-Assisted Dispersive Liquid-Liquid Microextraction of Parabens from Personal Care Products and Urine, Followed by High-Performance Liquid Chromatography. Turk. J. Pharm. Sci..

[44] Nezami R.A., Tehrani M.S., Faraji H., Husain S.W., Azar P.A. (2021). Strategies to improve the challenges of classic dispersive liquid-liquid microextraction for determination of the parabens in personal care products-One step closer to green analytical chemistry. J. Chromatogr. B.

[45] García-Córcoles M.T., Rodríguez-Gómez R., de Alarcón-Gómez B., Çipa M., Martín-Pozo L., Kauffmann J.-M., Zafra-Gómez A. (2019). Chromatographic Methods for the Determination of Emerging Contaminants in Natural Water and Wastewater Samples: A Review. Crit. Rev. Anal. Chem..

[46] Farahmandi M., Yamini Y., Baharfar M., Karami M. (2021). Dispersive Magnetic Solid Phase Microextraction on Microfluidic Systems for Extraction and Determination of Parabens. Anal. Chim. Acta.

[47] Silveira R.S., Rocha B.A., Rodrigues J.L., Barbosa F. (2020). Rapid, Sensitive and Simultaneous Determination of 16 Endocrine-Disrupting Chemicals (Parabens, Benzophenones, Bisphenols, and Triclocarban) in Human Urine Based on Microextraction by Packed Sorbent Combined with Liquid Chromatography Tandem Mass Spectrometry (MEPS-LC-MS/MS). Chemosphere.

[48] Serb A.F., Georgescu M., Onulov R., Novaconi C.R., Sisu E., Bolocan A., Sandu R.E. (2024). Mass-Spectrometry-Based Research of Cosmetic Ingredients. Molecules.

[49] Alqarni A.M. (2024). Analytical Methods for the Determination of Pharmaceuticals and Personal Care Products in Solid and Liquid Environmental Matrices: A Review. Molecules.

[50] Wang L., Yan X., Chen X., Li Y., Wu D. (2024). Magnetic Polyimide Nanocomposite for Analysis of Parabens in Cooking Wine by Magnetic Solid-Phase Extraction Coupled with Gas Chromatography–Mass Spectrometry. J. Chromatogr. A.

[51] Azzouz A., Colón L.P., Hejji L., Ballesteros E. (2020). Determination of Alkylphenols, Phenylphenols, Bisphenol A, Parabens, Organophosphorus Pesticides and Triclosan in Different Cereal-Based Foodstuffs by Gas Chromatography–Mass Spectrometry. Anal. Bioanal. Chem..

[52] Ali H.M., Alsohaimi I.H., Khan M.R., Azam M. (2020). Simultaneous Determination of Isothiazolinones and Parabens in Cosmetic Products Using Solid-Phase Extraction and Ultra-High Performance Liquid Chromatography/Diode Array Detector. Pharmaceuticals.

[53] Pasupuleti R.R., Hsieh J.-R., Pasupuleti V.R., Huang Y.-L. (2022). Eco-Friendly Magnetic Solid-Phase Extraction and Deep Eutectic Solvent for the Separation and Detection of Parabens from the Environmental Water and Urine Samples. Microchem. J..

[54] Nouri N., Khorram P., Duman O., Sibel T., Hassan S. (2020). Overview of nanosorbents used in solid phase extraction techniques for the monitoring of emerging organic contaminants in water and wastewater samples. Trends Environ. Anal. Chem..

[55] Alampanos V., Samanidou V. (2021). An Overview of Sample Preparation Approaches Prior to Liquid Chromatography Methods for the Determination of Parabens in Biological Matrices. Microchem. J..

[56] Rudi F. (2020). Review of Analytical Methods for Determination of Parabens in Cosmetic Products. Def. ST Tech. Bull..

[57] Badawy M.E.I., El-Nouby M.A.M., Kimani P.K., Lim L.W., Rabea E.I. (2022). A Review of the Modern Principles and Applications of Solid-Phase Extraction Techniques in Chromatographic Analysis. Anal. Sci..

[58] Ghorbani M., Aghamohammadhassan M., Ghorbani H., Zabihi A. (2020). Trends in Sorbent Development for Dispersive Micro-Solid Phase Extraction. Microchem. J..

[59] Muniandy Y., Mohamad S., Raoov M. (2024). Green and Efficient Magnetic Micro-Solid Phase Extraction Utilizing Tea Waste Impregnated with Magnetic Nanoparticles for the Analysis of Ibuprofen in Water Samples by Using UV-Vis Spectrophotometry. RSC Adv..

[60] Soares da Silva Burato J., Vargas Medina D.A., de Toffoli A.L., Vasconcelos Soares Maciel E., Mauro Lanças F. (2020). Recent Advances and Trends in Miniaturized Sample Preparation Techniques. J. Sep. Sci..

[61] Sekopelo A.G., Munonde T.S., Nqombolo A., Mpupa A., Nomngongo P.N. (2024). UiO-66@ Fe_3_O_4_ Nanocomposite as an Adsorbent in Dispersive Solid Phase Extraction of Metformin in Surface Water and Wastewater. Nano Express.

[62] Soleimani N., Rahimnejad M., Ezoji H. (2024). An overview of electrochemical sensing strategies for methylparaben analysis. J. Taiwan Inst. Chem. Eng..

[63] Ahmed H.E.H., Gumus Z.P., Soylak M. (2024). Determination of atrazine in food, water, and synthetic urine by activated carbon cloth (ACC) micro-solid-phase extraction (µSPE) with high-performance liquid chromatography–diode array detection (HPLC-DAD). Anal. Lett..

[64] Piao C., Chen L., Wang Y. (2014). A Review of the Extraction and Chromatographic Determination Methods for the Analysis of Parabens. J. Chromatogr. B.

[65] Phalipat P., Bunkoed O., Llompart M., Hongyok S., Nurerk P. (2024). Covalent organic framework composite hydrogel sorbent beads for vortex-assisted dispersive miniaturized solid phase extraction of parabens and synthetic phenolic antioxidants in foodstuffs. Microchem. J..

[66] Jebali S., Labidi A., Sghaier R.B., Latrous L., Megriche A. (2024). The potential of three different adsorbents in solid-phase extraction of antihistaminic and antimigraine drugs from water samples using ultra-high-performance liquid chromatography-ultraviolet analysis. Sep. Sci. Plus.

[67] Morin-Crini N., Lichtfouse E., Liu G., Balaram V., Ribeiro A.R.L., Lu Z., Stock F., Carmona E., Teixeira M.R., Picos-Corrales L.A. (2021). Emerging Contaminants: Analysis, Aquatic Compartments and Water Pollution. Emerging Contaminants Vol. 1.

[68] Barbosa M.O., Ribeiro R.S., Ribeiro A.R.L., Pereira M.F.R., Silva A.M.T. (2020). Solid-Phase Extraction Cartridges with Multi-Walled Carbon Nanotubes and Effect of the Oxygen Functionalities on the Recovery Efficiency of Organic Micropollutants. Sci. Rep..

[69] Plesnik H., Bosnjak M., Cemazar M., Sersa G., Kosjek T. (2023). An Effective Validation of Analytical Method for Determination of a Polar Complexing Agent: The Illustrative Case of Cytotoxic Bleomycin. Anal. Bioanal. Chem..

[70] Birolli W.G., Lanças F.M., dos Santos Neto Á.J., Silveira H.C. (2024). Determination of pesticide residues in urine by chromatography-mass spectrometry: Methods and applications. Front. Public Health.

[71] Płotka-Wasylka J., Chabowska A., Pantanit S., Bunkoed O., Fares M.Y., Sajid M., Lambropoulou D., Kurowska-Susdorf A., Jatkowska N. (2024). Natural/Bio-Based Sorbents as Greener Extractive Materials for Endocrine Disrupting Compounds in Samples of Different Matrix Composition. TrAC Trends Anal. Chem..

[72] Vieira C.M.S., Mafra G., Brognoli R., Richter P., Rosero-Moreano M., Carasek E. (2020). A High Throughput Approach to Rotating-Disk Sorptive Extraction (RDSE) Using Laminar Cork for the Simultaneous Determination of Multiclass Organic Micro-Pollutants in Aqueous Sample by GC-MS. Talanta.

[73] Carasek E., Scur R., Bernardi G. (2023). High-Throughput Analytical Methods Employing Microextraction Techniques: Towards Fast Analyses Lasting a Few Seconds. Adv. Sample Prep..

[74] Becerra-Herrera M., Miranda V., Arismendi D., Richter P. (2018). Chemometric Optimization of the Extraction and Derivatization of Parabens for Their Determination in Water Samples by Rotating-Disk Sorptive Extraction and Gas Chromatography Mass Spectrometry. Talanta.

[75] Gutiérrez-Serpa A., Allgaier-Díaz D.W., Jiménez-Abizanda A.I., Pino V. (2023). Green Sorbents in Analytical Chemistry. Green Approaches for Chemical Analysis.

[76] Sajid M. (2022). Chitosan-Based Adsorbents for Analytical Sample Preparation and Removal of Pollutants from Aqueous Media: Progress, Challenges and Outlook. Trends Environ. Anal. Chem..

[77] Mallek M. (2018). Analytical Methodology Based on a Silicone Rod (SR) Micro Extraction Combined with HPLC-DAD Method for the Determination of Pharmaceuticals and Antibacterial Products in Effluent Wastewaters: Characterization of the Sorption Removal Processes by Cork. Ph.D. Thesis.

[78] Richter P., Arismendi D., Becerra-Herrera M. (2021). The Fundamentals, Chemistries and Applications of Rotating-Disk Sorptive Extraction. TrAC Trends Anal. Chem..

[79] Hang N., Yang Y., Zang Y., Zhao W., Tao J., Li S. (2023). Magnetic cork composites as biosorbents in dispersive solid-phase extraction of pesticides in water samples. Anal. Methods.

[80] Manzo V., Goya-Pacheco J., Arismendi D., Becerra-Herrera M., Castillo-Aguirre A., Castillo-Felices R., Rosero-Moreano M., Carasek E., Richter P. (2019). Cork Sheet as a Sorptive Phase to Extract Hormones from Water by Rotating-Disk Sorptive Extraction (RDSE). Anal. Chim. Acta.

[81] Arismendi D., Becerra-Herrera M., Cerrato I., Richter P. (2019). Simultaneous Determination of Multiresidue and Multiclass Emerging Contaminants in Waters by Rotating-Disk Sorptive Extraction–Derivatization-Gas Chromatography/Mass Spectrometry. Talanta.

[82] Liu Z., Yang Y., Ye K., Duan Y., Wan Y., Shi X., Xu Z. (2024). Simultaneous and sensitive detection of methylparaben and its metabolites by using molecularly imprinted solid-phase microextraction fiber array technique. Anal. Chim. Acta.

[83] Casado-Carmona F.A., Lucena R., Cárdenas S. (2021). Magnetic Paper-Based Sorptive Phase for Enhanced Mass Transference in Stir Membrane Environmental Samplers. Talanta.

[84] Rojas-Candia V., Arismendi D., Richter P. (2022). Determination of Ibuprofen and 1-Hydroxyibuprofen in Aqueous Samples Using Cork as a Natural Phase in Rotating-Disk Sorptive Extraction. J. Chil. Chem. Soc..

[85] Rojas-Candia V., Arismendi D., Carasek E., Richter P. (2024). Cork-Activated Carbon as a Sorptive Phase for Microextraction of Emerging Contaminants in Water Samples. Braz. J. Anal. Chem..

[86] Torres-Lara N., Molina-Balmaceda A., Arismendi D., Richter P. (2023). Peanut Shell-Derived Activated Biochar as a Convenient, Low-Cost, Ecofriendly and Efficient Sorbent in Rotating Disk Sorptive Extraction of Emerging Contaminants from Environmental Water Samples. Green. Anal. Chem..

[87] Vieira C.M.S., Mazurkievicz M., Lopez Calvo A.M., Debatin V., Micke G.A., Richter P., Rosero-Moreano M., Rocha E.C. (2018). da Exploiting Green Sorbents in Rotating-disk Sorptive Extraction for the Determination of Parabens by High-performance Liquid Chromatography with Tandem Electrospray Ionization Triple Quadrupole Mass Spectrometry. J. Sep. Sci..

[88] Kannouma R.E., Hammad M.A., Kamal A.H., Mansour F.R. (2022). Miniaturization of Liquid-Liquid Extraction; the Barriers and the Enablers. Microchem. J..

[89] Sajid M. (2022). Dispersive Liquid-Liquid Microextraction: Evolution in Design, Application Areas, and Green Aspects. TrAC Trends Anal. Chem..

[90] Khatibi S.A., Hamidi S., Siahi-Shadbad M.R. (2022). Application of Liquid-Liquid Extraction for the Determination of Antibiotics in the Foodstuff: Recent Trends and Developments. Crit. Rev. Anal. Chem..

[91] Câmara J.S., Perestrelo R., Olayanju B., Berenguer C.V., Kabir A., Pereira J.A.M. (2022). Overview of Different Modes and Applications of Liquid Phase-Based Microextraction Techniques. Processes.

[92] Loh S.H., Yahaya N., Ishak S.M., Wan Mohd Khalik W.M.A., Che Abdullah N.S., Aboul-Enein H.Y., Ong M.C. (2021). Recent Trends in Adsorbent-Based Microextraction of Micropollutants in Environmental Waters. Curr. Pollut. Rep..

[93] El-Deen A.K. (2024). An Overview of Recent Advances and Applications of Matrix Solid-Phase Dispersion. Sep. Purif. Rev..

[94] Saleh N.M., Hafiz N.M., NikWee N.N.A. (2020). Determination of Parabens from Water Samples Using Cloud Point Extraction, Vortex Extraction and Liquid–Liquid Extraction Method Coupled with High Performance Liquid Chromatography. J. Comput. Theor. Nanosci..

[95] Beh S.Y., Mahfut I., Juber N., Asman S., Yusoff F., Saleh N.M. (2021). Extraction of Parabens from Cosmetic and Environmental Water Samples Coupled with UV-Visible Spectroscopy. J. Appl. Spectrosc..

[96] Mostafa A., Shaaban H., Alqarni A.M., Alghamdi M., Alsultan S., Al-Saeed J.S., Alsaba S., AlMoslem A., Alshehry Y., Ahmad R. (2022). Vortex-Assisted Dispersive Liquid–Liquid Microextraction Using Thymol Based Natural Deep Eutectic Solvent for Trace Analysis of Sulfonamides in Water Samples: Assessment of the Greenness Profile Using AGREE Metric, GAPI and Analytical Eco-Scale. Microchem. J..

[97] Abdussalam-Mohammed W., Ali A.Q., Errayes A.O. (2020). Green Chemistry: Principles, Applications, and Disadvantages. Chem. Methodol..

[98] Liao F.-Y., Su Y.-L., Weng J.-R., Lin Y.-C., Feng C.-H. (2020). Ultrasound–Vortex-Assisted Dispersive Liquid–Liquid Microextraction Combined with High Performance Liquid Chromatography–Diode Array Detection for Determining UV Filters in Cosmetics and the Human Stratum Corneum. Molecules.

[99] Dalmaz A., Özak S.S. (2022). DES-Based Vortex-Assisted Liquid-Liquid Microextraction Procedure Developed for the Determination of Paraben Preservatives in Mouthwashes. Microchem. J..

[100] Pérez-Lemus N., López-Serna R., Pérez-Elvira S.I., Barrado E. (2020). Sample Pre-Treatment and Analytical Methodology for the Simultaneous Determination of Pharmaceuticals and Personal Care Products in Sewage Sludge. Chemosphere.

[101] Bocelli M.D., Vargas Medina D.A., Rodriguez J.P., Lanças F.M., Santos-Neto Á.J. (2022). Determination of parabens in wastewater samples via robot-assisted dynamic single-drop microextraction and liquid chromatography–tandem mass spectrometry. Electrophoresis.

[102] Mei M., Pang J., Huang X. (2018). Development of a sensitive method for the determination of parabens in complex samples by online coupling of magnetism-enhanced monolith-based in-tube solid phase microextraction with high performance liquid chromatography. Anal. Methods.

[103] Fenni F., Sunyer-Caldú A., Ben Mansour H., Diaz-Cruz M.S. (2022). Contaminants of Emerging Concern in Marine Areas: First Evidence of UV Filters and Paraben Preservatives in Seawater and Sediment on the Eastern Coast of Tunisia. Environ. Pollut..

[104] Yang Y., Ok Y.S., Kim K.-H., Kwon E.E., Tsang Y.F. (2017). Occurrences and Removal of Pharmaceuticals and Personal Care Products (PPCPs) in Drinking Water and Water/Sewage Treatment Plants: A Review. Sci. Total Environ..

[105] Mpayipheli N., Mpupa A., Madala N.E., Nomngongo P.N. (2024). Paraben Residues in Wastewater and Surface Water: A Case Study of KwaZulu Natal and Gauteng Provinces (South Africa) during the COVID-19 Pandemic. Front. Environ. Sci..

[106] Gumbi B.P., Moodley B., Birungi G., Ndungu P.G. (2022). Risk Assessment of Personal Care Products, Pharmaceuticals, and Stimulants in Mgeni and Msunduzi Rivers, KwaZulu-Natal, South Africa. Front. Water.

[107] Mashile G.P., Mpupa A., Nomngongo P.N. (2021). Magnetic Mesoporous Carbon/β-Cyclodextrin–Chitosan Nanocomposite for Extraction and Preconcentration of Multi-Class Emerging Contaminant Residues in Environmental Samples. Nanomaterials.

[108] Muckoya V.A., Nomngongo P.N., Ngila J.C. (2020). Factorial Design Optimisation of Solid Phase Extraction for Preconcentration of Parabens in Wastewater Using Ultra-High Performance Liquid Chromatography Triple Quadrupole Mass Spectrometry. Curr. Anal. Chem..

[109] Ngila J., Matheri A., Muckoya V., Ngigi E., Ntuli F., Seodigeng T., Zvinowanda C. (2020). Mathematical Modelling for Biological Wastewater Treatment Plants Gauteng, South Africa.

[110] Jakavula S., Nqombolo A., Mpupa A., Ren J., Nomngongo P.N. (2023). Hybrid Porous Material Supported in a Cellulose Acetate Polymeric Membrane for the Direct Immersion Thin-Film Microextraction of Parabens in Water. J. Chromatogr. A.

[111] Archer E., Petrie B., Kasprzyk-Hordern B., Wolfaardt G.M. (2017). The Fate of Pharmaceuticals and Personal Care Products (PPCPs), Endocrine Disrupting Contaminants (EDCs), Metabolites and Illicit Drugs in a WWTW and Environmental Waters. Chemosphere.

[112] Archer E., Wolfaardt G.M., Tucker K.S. (2020). Substances of Emerging Concern in South African Aquatic Ecosystems.

[113] Galinaro C.A., Spadoto M., de Aquino F.W.B., de Souza Pelinson N., Vieira E.M. (2022). Environmental Risk Assessment of Parabens in Surface Water from a Brazilian River: The Case of Mogi Guaçu Basin, São Paulo State, under Precipitation Anomalies. Environ. Sci. Pollut. Res..

[114] Becerra-Herrera M., Miranda V., Richter P. (2020). Rapid Determination of Parabens in Water Samples by Ultra-High Performance Liquid Chromatography Coupled to Time of Flight Mass Spectrometry. Anal. Sci..

[115] Saha S., Narayanan N., Singh N., Gupta S. (2022). Occurrence of Endocrine Disrupting Chemicals (EDCs) in River Water, Ground Water and Agricultural Soils of India. Int. J. Environ. Sci. Technol..

[116] Feng J., Zhao J., Xi N., Guo W., Sun J. (2019). Parabens and Their Metabolite in Surface Water and Sediment from the Yellow River and the Huai River in Henan Province: Spatial Distribution, Seasonal Variation and Risk Assessment. Ecotoxicol. Environ. Saf..

[117] Arfaeinia H., Asadgol Z., Ramavandi B., Dobaradaran S., Kalantari R.R., Poureshgh Y., Behroozi M., Asgari E., Asl F.B., Sahebi S. (2022). Monitoring and Eco-Toxicity Effect of Paraben-Based Pollutants in Sediments/Seawater, North of the Persian Gulf. Env. Geochem. Health.

[118] K’oreje K.O., Vergeynst L., Ombaka D., De Wispelaere P., Okoth M., Van Langenhove H., Demeestere K. (2016). Occurrence Patterns of Pharmaceutical Residues in Wastewater, Surface Water and Groundwater of Nairobi and Kisumu City, Kenya. Chemosphere.

[119] K’oreje K.O., Kandie F.J., Vergeynst L., Abira M.A., Van Langenhove H., Okoth M., Demeestere K. (2018). Occurrence, Fate and Removal of Pharmaceuticals, Personal Care Products and Pesticides in Wastewater Stabilization Ponds and Receiving Rivers in the Nzoia Basin, Kenya. Sci. Total Environ..

[120] Bolujoko N.B., Olorunnisola D., Poudel S., Omorogie M.O., Ogunlaja O.O., Olorunnisola C.G., Adesina M., Deguenon E., Dougnon V., Alfred M.O. (2024). Occurrence Profiling, Risk Assessment, and Correlations of Antimicrobials in Surface Water and Groundwater Systems in Southwest Nigeria. Env. Sci. Process Impacts.

[121] Le T.M., Pham P.T., Nguyen T.Q., Nguyen T.Q., Bui M.Q., Nguyen H.Q., Vu N.D., Kannan K., Tran T.M. (2022). A survey of parabens in aquatic environments in Hanoi, Vietnam and its implications for human exposure and ecological risk. Environ. Sci. Pollut. Res..

[122] Czarczyńska-Goślińska B., Zgoła-Grześkowiak A., Jeszka-Skowron M., Frankowski R., Grześkowiak T. (2017). Detection of Bisphenol A, Cumylphenol and Parabens in Surface Waters of Greater Poland Voivodeship. J. Env. Manag..

[123] Adhikari S., Kumar R., Driver E.M., Perleberg T.D., Yanez A., Johnston B., Halden R.U. (2022). Mass trends of parabens, triclocarban and triclosan in Arizona wastewater collected after the 2017 FDA ban on antimicrobials and during the COVID-19 pandemic. Water Res..

[124] Rojo M., Ball A.L., Penrose M.T., Weir S.M., LeBaron H., Terasaki M., Cobb G.P., Lavado R. (2024). Accumulation of Parabens, Their Metabolites, and Halogenated Byproducts in Migratory Birds of Prey: A Comparative Study in Texas and North Carolina, USA. Environ. Toxicol. Chem..

[125] Albouy M., Deceuninck Y., Migeot V., Doumas M., Dupuis A., Venisse N., Engene P.P., Veyrand B., Geny T., Marchand P. (2023). Characterization of Pregnant Women Exposure to Halogenated Parabens and Bisphenols through Water Consumption. J. Hazard. Mater..

[126] Emnet P., Mahaliyana A.S., Northcott G., Gaw S. (2020). Organic Micropollutants in Wastewater Effluents and the Receiving Coastal Waters, Sediments, and Biota of Lyttelton Harbour (Te Whakaraupō), New Zealand. Arch. Environ. Contam. Toxicol..

[127] Gani K.M., Hlongwa N., Abunama T., Kumari S., Bux F. (2021). Emerging Contaminants in South African Water Environment- a Critical Review of Their Occurrence, Sources and Ecotoxicological Risks. Chemosphere.

[128] Yang C.W., Lee W.C. (2023). Parabens increase sulfamethoxazole-, tetracycline-and paraben-resistant bacteria and reshape the nitrogen/sulfur cycle-associated microbial communities in freshwater river sediments. Toxics.

[129] Dalmaz A., Sivrikaya Özak S. (2022). Development of Clinoptilolite Zeolite-Coated Magnetic Nanocomposite-Based Solid Phase Microextraction Method for the Determination of Rhodamine B in Cosmetic Products. J. Chromatogr. A.

[130] Moreno-Marenco A.R., Giraldo L., Moreno-Piraján J.C. (2019). Parabens Adsorption onto Activated Carbon: Relation with Chemical and Structural Properties. Molecules.

[131] Nodeh H.R., Sereshti H., Ataolahi S., Toloutehrani A., Ramezani A.T. (2020). Activated Carbon Derived from Pistachio Hull Biomass for the Effective Removal of Parabens from Aqueous Solutions: Isotherms, Kinetics, and Free Energy Studies. Desalination Water Treat..

[132] Guo M., Chen W., Ru H., Yang L., Zhang Y. (2020). Adsorption Characteristics of Ethyl Paraben from Aqueous Solution Using Rice Husk Biochar. Proceedings of the IOP Conference Series: Materials Science and Engineering.

[133] Mashile G.P., Mpupa A., Nqombolo A., Dimpe K.M., Nomngongo P.N. (2020). Recyclable Magnetic Waste Tyre Activated Carbon-Chitosan Composite as an Effective Adsorbent Rapid and Simultaneous Removal of Methylparaben and Propylparaben from Aqueous Solution and Wastewater. J. Water Process Eng..

[134] González-Hernández P., Gutiérrez-Serpa A., Lago A.B., Estévez L., Ayala J.H., Pino V., Pasán J. (2021). Insights into Paraben Adsorption by Metal-Organic Frameworks for Analytical Applications. ACS Appl. Mater. Interfaces.

[135] Pourmohammad M., Ghadi A., Beni A.A. (2024). Removal of Benzyl Paraben from Wastewater Using Zeolitic Imidazolate-67 Modified by Fe_3_O_4_ Nanoparticles with Response Surface Methodology. J. Appl. Chem. Res..

[136] Correa-Navarro Y.M., Rivera-Giraldo J.D., Cardona-Castaño J.A. (2024). Modified Cellulose for Adsorption of Methylparaben and Butylparaben from an Aqueous Solution. ACS Omega.

[137] Cosano D., Esquivel D., Romero-Salguero F.J., Jiménez-Sanchidrián C., Ruiz J.R. (2023). Carboxymethylcellulose/Hydrotalcite Bionanocomposites as Paraben Sorbents. Langmuir.

[138] Pourmohammad M., Ghadi A., Beni A.A. (2023). Response Surface Methodology for Adsorption of Propylparaben Using Zeolitic Imidazolate-67 Modified by Fe3 O4 Nanoparticles from Aqueous Solutions. Desalination Water Treat..

[139] León G., Hidalgo A.M., Martínez A., Guzmán M.A., Miguel B. (2023). Methylparaben Adsorption onto Activated Carbon and Activated Olive Stones: Comparative Analysis of Efficiency, Equilibrium, Kinetics and Effect of Graphene-Based Nanomaterials Addition. Appl. Sci..

[140] Chen H.W., Chiou C.S., Chang S.H. (2017). Comparison of Methylparaben, Ethylparaben and Propylparaben Adsorption onto Magnetic Nanoparticles with Phenyl Group. Powder Technol..

[141] Pargari M., Marahel F., Godajdar B.M. (2021). Ultrasonic-Assisted Adsorption of Propyl Paraben on Ultrasonically Synthesized TiO_2_ Nanoparticles Loaded on Activated Carbon: Optimization, Kinetic and Equilibrium Studies. Desalination Water Treat..

[142] Gwenzi W., Chaukura N., Noubactep C., Mukome F.N.D. (2017). Biochar-Based Water Treatment Systems as a Potential Low-Cost and Sustainable Technology for Clean Water Provision. J. Environ. Manag..

[143] Adam M.R., Othman M.H.D., Kurniawan T.A., Puteh M.H., Ismail A.F., Khongnakorn W., Rahman M.A., Jaafar J. (2022). Advances in Adsorptive Membrane Technology for Water Treatment and Resource Recovery Applications: A Critical Review. J. Environ. Chem. Eng..

[144] Salehi Nasab F., Ahmadi Azqhandi M.H., Ghalami-Choobar B. (2024). Evaluating the Efficacy of Recyclable Nanostructured Adsorbents for Rapid Removal of Methylparaben from Aqueous Solutions. Environ. Res..

[145] Almeida-Naranjo C.E., Guerrero V.H., Villamar-Ayala C.A. (2023). Emerging contaminants and their removal from aqueous media using conventional/non-conventional adsorbents: A glance at the relationship between materials, processes, and technologies. Water.

[146] Babu Ponnusami A., Sinha S., Ashokan H., V Paul M., Hariharan S.P., Arun J., Gopinath K.P., Hoang Le Q., Pugazhendhi A. (2023). Advanced Oxidation Process (AOP) Combined Biological Process for Wastewater Treatment: A Review on Advancements, Feasibility and Practicability of Combined Techniques. Environ. Res..

[147] Shi H., Ni J., Zheng T., Wang X., Wu C., Wang Q. (2020). Remediation of Wastewater Contaminated by Antibiotics. A Review. Environ. Chem. Lett..

[148] Karić N., Maia A.S., Teodorović A., Atanasova N., Langergraber G., Crini G., Ribeiro A.R.L., Đolić M. (2022). Bio-Waste Valorisation: Agricultural Wastes as Biosorbents for Removal of (in)Organic Pollutants in Wastewater Treatment. Chem. Eng. J. Adv..

[149] Lincho J., Gomes J., Kobylanski M., Bajorowicz B., Zaleska-Medynska A., Martins R.C. (2021). TiO_2_ Nanotube Catalysts for Parabens Mixture Degradation by Photocatalysis and Ozone-Based Technologies. Process Saf. Environ. Prot..

[150] Álvarez M.A., Ruidíaz-Martínez M., Cruz-Quesada G., López-Ramón M.V., Rivera-Utrilla J., Sánchez-Polo M., Mota A.J. (2020). Removal of Parabens from Water by UV-Driven Advanced Oxidation Processes. Chem. Eng. J..

[151] Gmurek M., Rossi A.F., Martins R.C., Quinta-Ferreira R.M., Ledakowicz S. (2015). Photodegradation of Single and Mixture of Parabens—Kinetic, by-Products Identification and Cost-Efficiency Analysis. Chem. Eng. J..

[152] Hu C., Tian M., Wu L., Chen L. (2022). Enhanced Photocatalytic Degradation of Paraben Preservative over Designed G-C3N4/BiVO4 S-Scheme System and Toxicity Assessment. Ecotoxicol. Environ. Saf..

[153] Fernandes E., Mazierski P., Klimczuk T., Zaleska-Medynska A., Martins R.C., Gomes J. (2023). G-C3N4 for Photocatalytic Degradation of Parabens: Precursors Influence, the Radiation Source and Simultaneous Ozonation Evaluation. Catalysts.

[154] Fernandes R.A., Sampaio M.J., Dražić G., Faria J.L., Silva C.G. (2020). Efficient Removal of Parabens from Real Water Matrices by a Metal-Free Carbon Nitride Photocatalyst. Sci. Total Environ..

[155] Ma J., Yang S., Shi H., Pang J., Zhang X., Wang Y., Sun H. (2020). An Efficient and Robust Exfoliated Bentonite/Ag3PO4/AgBr Plasmonic Photocatalyst for Degradation of Parabens. RSC Adv..

[156] Wei Y., Liu X., Wang Z., Chi Y., Yue T., Dai Y., Zhao J., Xing B. (2022). Adsorption and Catalytic Degradation of Preservative Parabens by Graphene-Family Nanomaterials. Sci. Total Environ..

[157] Huo Y., Li M., An Z., Jiang J., Zhou Y., Ma Y., Xie J., Wei F., He M. (2024). Effect of PH on UV/H_2_O_2_-Mediated Removal of Single, Mixed and Halogenated Parabens from Water. J. Hazard. Mater..

[158] Mohammadi L., Rahdar A., Bazrafshan E., Dahmardeh H., Susan M.A.B.H., Kyzas G.Z. (2020). Petroleum Hydrocarbon Removal Fromwastewaters: A Review. Processes.

[159] Nabavi E., Niavol K.P., Dezvareh G.A., Darban A.K. (2023). A Combined Treatment System of O_3_/UV Oxidation and Activated Carbon Adsorption: Emerging Contaminants in Hospital Wastewater. J. Water Health.

[160] Ncube A., Mtetwa S., Bukhari M., Fiorentino G., Passaro R. (2023). Circular Economy and Green Chemistry: The Need for Radical Innovative Approaches in the Design for New Products. Energy.

[161] Zbair M., Limousy L., Drané M., Richard C., Juge M., Aemig Q., Trably E., Escudié R., Peyrelasse C., Bennici S. (2024). Integration of Digestate-Derived Biochar into the Anaerobic Digestion Process through Circular Economic and Environmental Approaches—A Review. Materials.

[162] Tawalbeh M., Al Mojjly A., Al-Othman A., Hilal N. (2018). Membrane Separation as a Pre-Treatment Process for Oily Saline Water. Desalination.

[163] Chang H., Zhu Y., Yu H., Qu F., Zhou Z., Li X., Yang Y., Tang X., Liang H. (2022). Long-Term Operation of Ultrafiltration Membrane in Full-Scale Drinking Water Treatment Plants in China: Characteristics of Membrane Performance. Desalination.

[164] Shinde P.A., Ukarde T.M., Gogate P.R., Pawar H.S. (2021). An Integrated Approach of Adsorption and Membrane Separation for Treatment of Sewage Water and Resource Recovery. J. Water Process Eng..

[165] Bairagi S., Ali S.W. (2020). Conventional and Advanced Technologies for Wastewater Treatment. Environmental Nanotechnology for Water Purification.

[166] López-Ortiz C.M., Sentana-Gadea I., Varó-Galvañ P., Maestre-Pérez S.E., Prats-Rico D. (2018). The Use of Combined Treatments for Reducing Parabens in Surface Waters: Ion-Exchange Resin and Nanofiltration. Sci. Total Environ..

[167] Bhattacharya P., Mukherjee D., Deb N., Swarnakar S., Banerjee S. (2021). Indigenously Developed CuO/TiO_2_ Coated Ceramic Ultrafiltration Membrane for Removal of Emerging Contaminants like Phthalates and Parabens: Toxicity Evaluation in PA-1 Cell Line. Mater. Chem. Phys..

[168] Kohli H.P., Gupta S., Chakraborty M. (2020). Characterization and Stability Study of Pseudo-Emulsion Hollow Fiber Membrane: Separation of Ethylparaben. Colloids Surf. A Physicochem. Eng. Asp..

[169] Khoo Y.S., Goh P.S., Lau W.J., Ismail A.F., Abdullah M.S., Mohd Ghazali N.H., Yahaya N.K.E.M., Hashim N., Othman A.R., Mohammed A. (2022). Removal of Emerging Organic Micropollutants via Modified-Reverse Osmosis/Nanofiltration Membranes: A Review. Chemosphere.

[170] Osman A.I., Chen Z., Elgarahy A.M., Farghali M., Mohamed I.M.A., Priya A.K., Hawash H.B., Yap P.S. (2024). Membrane Technology for Energy Saving: Principles, Techniques, Applications, Challenges, and Prospects. Adv. Energy Sustain. Res..

[171] Díez B., Rosal R. (2020). A Critical Review of Membrane Modification Techniques for Fouling and Biofouling Control in Pressure-Driven Membrane Processes. Nanotechnol. Environ. Eng..

[172] Yin J., Zhang H. (2021). fang A Combined Physical Blending and Surface Grafting Strategy for Hydrophilic Modification of Polyethersulfone Membrane toward Oil/Water Separation. Polymer.

[173] Alnajjar H., Tabatabai A., Alpatova A., Leiknes T., Ghaffour N. (2021). Organic Fouling Control in Reverse Osmosis (RO) by Effective Membrane Cleaning Using Saturated CO2 Solution. Sep. Purif. Technol..

[174] Song J., Yan M., Ye J., Zheng S., Ee L.Y., Wang Z., Li J., Huang M. (2022). Research Progress in External Field Intensification of Forward Osmosis Process for Water Treatment: A Critical Review. Water Res..

[175] Hidalgo A.M., León G., Murcia M.D., Gómez M., Gómez E., Gómez J.L. (2021). Using Pressure-Driven Membrane Processes to Remove Emerging Pollutants from Aqueous Solutions. Int. J. Environ. Res. Public. Health.

[176] Shirasangi R., Kohli H.P., Gupta S., Chakraborty M. (2020). Separation of Methylparaben by Emulsion Liquid Membrane: Optimization, Characterization, Stability and Multiple Cycles Studies. Colloids Surf. A Physicochem. Eng. Asp..

[177] Ngigi E.M., Nomngongo P.N., Ngila J.C. (2022). Recent Methods Used in Degradation of Parabens in Aqueous Solutions: A Review. Int. J. Environ. Sci. Technol..

[178] Rita Pereira A., Gomes I.B., Harir M., Santos L., Simões M. (2024). Parabens Transformation Products in Water and Their (Eco)Toxicological Implications. Chem. Eng. J..

[179] Surana D., Gupta J., Sharma S., Kumar S., Ghosh P. (2022). A Review on Advances in Removal of Endocrine Disrupting Compounds from Aquatic Matrices: Future Perspectives on Utilization of Agri-Waste Based Adsorbents. Sci. Total Environ..

[180] Somma S., Reverchon E., Baldino L. (2021). Water Purification of Classical and Emerging Organic Pollutants: An Extensive Review. ChemEngineering.

[181] Penrose M.T., Cobb G.P. (2022). Identifying Potential Paraben Transformation Products and Evaluating Changes in Toxicity as a Result of Transformation. Water Environ. Res..

[182] Fransway A.F., Fransway P.J., Belsito D.V., Warshaw E.M., Sasseville D., Fowler J.F., DeKoven J.G., Pratt M.D., Maibach H.I., Taylor J.S. (2019). Parabens. Dermatitis.

[183] Dhangar K., Kumar M. (2020). Tricks and Tracks in Removal of Emerging Contaminants from the Wastewater through Hybrid Treatment Systems: A Review. Sci. Total Environ..

[184] Liu T., Aniagor C.O., Ejimofor M.I., Menkiti M.C., Tang K.H.D., Chin B.L.F., Chan Y.H., Yiin C.L., Cheah K.W., Ho Chai Y. (2023). Technologies for Removing Pharmaceuticals and Personal Care Products (PPCPs) from Aqueous Solutions: Recent Advances, Performances, Challenges and Recommendations for Improvements. J. Mol. Liq..

[185] Zhou H., Wei C., Zhang F., Liao J., Hu Y., Wu H. (2018). Energy-Saving Optimization of Coking Wastewater Treated by Aerobic Bio-Treatment Integrating Two-Stage Activated Carbon Adsorption. J. Clean. Prod..

[186] Lefatle M.C., Matong J.M., Mpupa A., Munonde T.S., Waleng N.J., Madikizela L.M., Pakade V.E., Nomngongo P.N. (2023). Preparation, Characterization, and Application of Chitosan–Kaolin-Based Nanocomposite in Magnetic Solid-Phase Extraction of Tetracycline in Aqueous Samples. Chem. Pap..

[187] Mashile P.P., Munonde T.S., Nomngongo P.N. (2023). Occurrence and Adsorptive Removal of Sulfonamides and β-Blockers in African and Asian Water Matrices: A Comprehensive Review. Environ. Adv..

[188] Tekin Z., Karlıdağ N.E., Özdoğan N., Koçoğlu E.S., Bakırdere S. (2022). Dispersive Solid Phase Extraction Based on Reduced Graphene Oxide Modified Fe3O4 Nanocomposite for Trace Determination of Parabens in Rock, Soil, Moss, Seaweed, Feces, and Water Samples from Horseshoe and Faure Islands. J. Hazard. Mater..

[189] Jiang Y., Bian X., Zhang M., Zhang H., Yu K., Kan G., Feng Y., Wang X., Song D., Jiang J. (2023). Application of Dispersive Liquid–Liquid Extraction Followed by Rapid and Direct Mass Spectrometry Analysis to Evaluate Parabens in High Salinity Water Sample. Microchem. J..

[190] UNION P. (2009). Regulation (EC) No 1223/2009 of the European Parliament and of the Council. Off. J. Eur. Union L.

[191] Nowak K., Ratajczak–Wrona W., Górska M., Jabłońska E. (2018). Parabens and Their Effects on the Endocrine System. Mol. Cell Endocrinol..

[192] Mishra L., Kurmi B. (2023). Das Cosmetics Regulations and Standardization Guidelines. Pharmaspire.

[193] Pollock T., Karthikeyan S., Walker M., Werry K., St-Amand A. (2021). Trends in Environmental Chemical Concentrations in the Canadian Population: Biomonitoring Data from the Canadian Health Measures Survey 2007–2017. Environ. Int..

[194] Khan N.S. (2023). Childhood and Adolescent Exposure to Chemicals Found in Personal Care Products. Master’s Thesis.

[195] Cabaleiro N., De La Calle I., Bendicho C., Lavilla I. (2014). An Overview of Sample Preparation for the Determination of Parabens in Cosmetics. TrAC Trends Anal. Chem..

