# Supplementary material for: Review of the Integrated Approaches for Monitoring and Treating Parabens in Water Matrices
Source: Molecules. 2024 Nov 22;29(23):5533. doi: 10.3390/molecules29235533 (PMC11643543; doi:10.3390/molecules29235533)
Supplement: Supplementary file 1 [file molecules-29-05533-s001.zip › molecules-3277412-supplementary.pdf]

molecules

Review

Review of the Integrated Approaches for Monitoring and Treating Parabens in Water Matrices

Denga Ramutshatsha-Makhwedzha * and Tshimangadzo S. Munonde *

Institute for Nanotechnology and Water Sustainability, College of Science, Engineering, and Technology, University of South Africa, Florida Science Campus, Roodepoort 1710, South Africa
* Correspondence: ramumd@unisa.ac.za (D.R.-M.); munonts@unisa.ac.za (T.S.M.); Tel.: +27-11-670-9848 (D.R.-M)

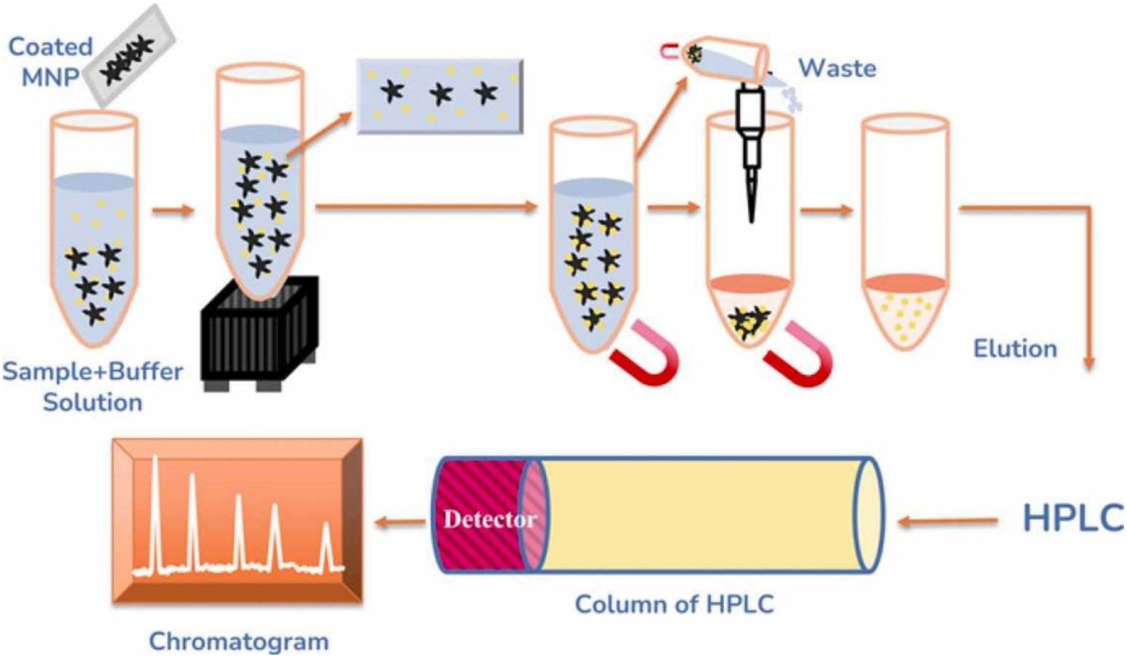

Figure S1 Fe_3O_4@rGO-DSPE method was combined with HPLC-UV for trace paraben determination. Reproduced with permission from [1].

Tekin et al. [1] developed a dispersive solid phase extraction method combined with HPLC-UV for the trace determination of methyl paraben, ethyl paraben, propyl paraben, butylparaben, and benzyl paraben in a variety of sample matrices. Taking advantage of the magnetic properties of the Fe_3O_4@rGO nanocomposite, the authors were able to simultaneously preconcentrate the trace parabens using an ultrasound-supported dispersive solid phase extraction (DSPE) method. This allowed for the easy recovery of the adsorbent materials via applying a magnetic field, enabling the repetition of many preconcentration cycles. Furthermore, the use of magnetic materials eliminated a centrifugation or filtration step leading to a fast separation process, whilst achieving high selectivity and reducing interferences. Thus, the optimized Fe_3O_4@rGO-DSPE combined with HPLC-UV resulted in ultralow LOD in the range 0.02 – 0.16 ng mL^{-1}, R^2 in the range 0.9973 to 0.9998 yielding high recoveries in the range 87 – 117% on spiked seawater. The separation of the paraben analytes was achieved on the optimized isocratic program of 20 mM phosphate buffer (pH 4.50) and acetonitrile (58:42, v/v) that were used to elute and separate the analytes for detection on a reversed-phase C18 column. Due to the integrated DSPE preconcentration method with HPLC-UV, the authors were able to improve the sensitivity of the HPLC-UV system by about 15–48 folds. It is noteworthy that the preconcentration step is critical for

achieving high sensitivities, as well as the separation of analytes during the chromatographic analysis.

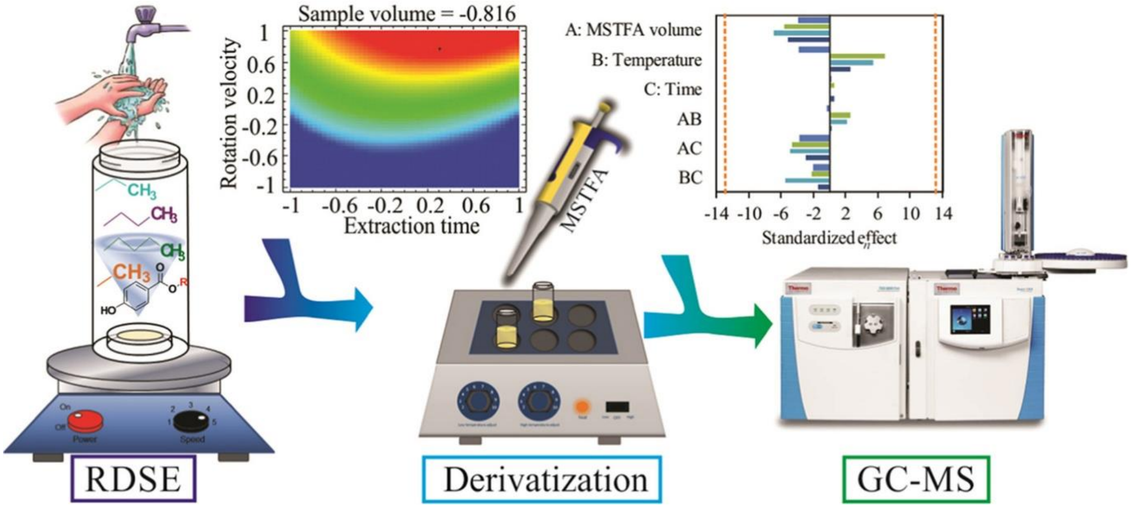

Figure S2 Extraction of parabens for their determination in water samples by rotating-disk sorptive extraction. Reproduced with permission from [2]

Becerra-Herrera et al. [2] studied the application of RDSE combined with GC-MS for the extraction and analysis of parabens as shown in Figure S2. The rapid extraction using RDSE resulted in the simultaneous determination of 4 parabens using GC-MS at 13 min. The detection limits were lower than 0.05 μg/L and recoveries studied using effluent samples of a wastewater treatment plant (WWTP) were higher than 80%. The main advantage of the RDSE method is faster extraction times and requires a considerably lower volume. Interestingly, the study combined RDSE with chemometric analysis that helped develop an environmentally friendly and economical method, thus reducing the number of analyses with the concomitant reduction of time, reagents, generated waste, and reduced cost. The challenge of the RDSE combined GC-MS analysis of parabens was the repeatability of the process as the %RSD was in most cases fluctuating to between 5-10%. This might require further analysis to ensure that the RDSE method is both reproducible and repeatable, whilst keeping the high sensitivity and selectivity.

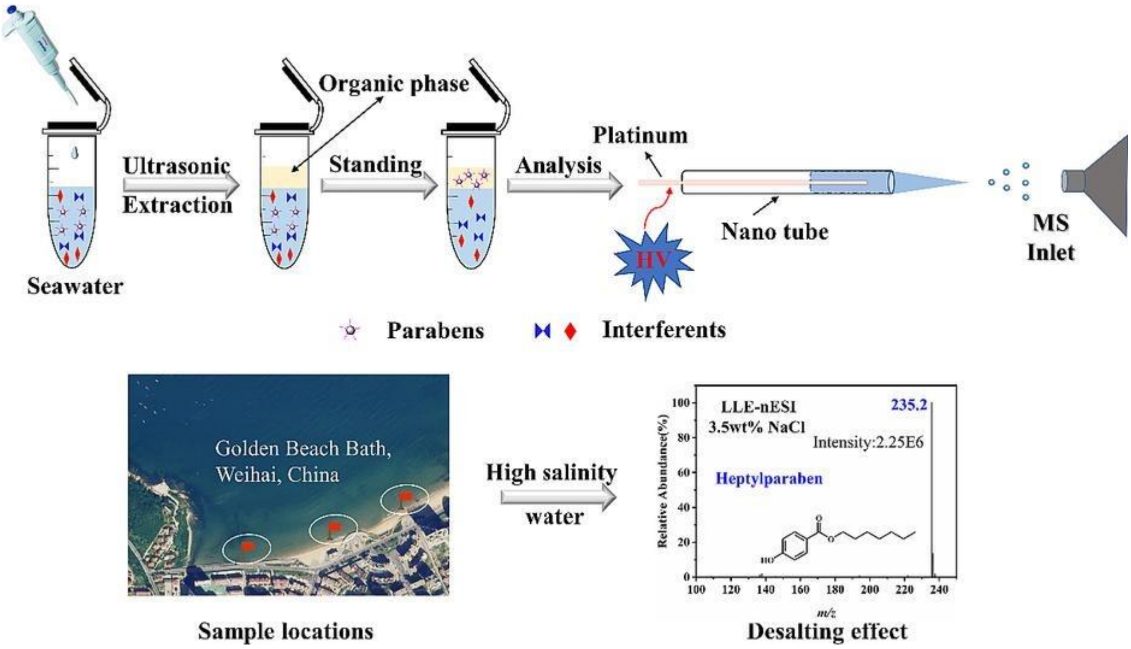

Figure S3 Sensitive and accurate ultrasound-assisted liquid-liquid extraction (UA-LLE) method for heptyl paraben extraction in saline water. Reproduced with permission from [3].

Figure S3 shows the application of the ultrasound-assisted liquid-liquid extraction (UA-LLE) combined with LC-MS on the extraction and analysis of heptyl paraben (HePB) in highly saline water. The authors reported that the extraction process requires only one organic phase and a small amount to perform effective desalting and improve the analyte response. The sensitivity was enhanced by using the nano-electrospray ionization method on the LC-MS, reducing the resident time. The UA-LLE extraction of HePB was reported to effectively reduce the interference of salt ions in saline water to avoid damaging the instrument. Compared to other contemporary methods such as SPE/LC-MS or RDSE/LC-MS, the UA-LLE/LC-MS was costly and not highly effective in the complete extraction of parabens.

Section S1: Policy and regulations of parabens

Parabens are commonly detected in environmental water matrices such as surface water, drinking water, and wastewater from wastewater treatment plants, thus, raising concerns due to their potential health and environmental impacts [3]. Several paraben compounds and their metabolites have been shown to have both estrogenic and anti-androgenic effects in vitro and in vivo, leading to their regulations and moratoriums in some countries [1]. **Table S1** shows the policies and regulations of parabens in products globally, with the lack of regulations, particularly in most African countries visualized. As seen in **Table S1**, the European Union and the U.S. Food and Drug Administration (FDA) have developed similar regulations for parabens in cosmetic products to monitor the safety of parabens in cosmetics. However, these regulations are not strict enough as concentrations of some parabens as high as ug/L are still detected in wastewater. Worryingly, there are currently no current regulations in the European Union and US-FDA that address the presence of parabens in water or wastewater. The EU and US-FDA do not consider parabens as water contaminants and have not set any water-related regulations, as the current restrictions are related to cosmetic products. Though the regulation on the use of parabens varies across different geographic regions as shown in **Table S1**, just like in the EU/US FDA, there are currently no regulations in water/wastewater.

Table S1 Policy regulations of parabens in various countries

Region	Maximum concentration	Regulation details	Comment	Ref
**Europe**	0.4% (as acid) for single ester, 0.8% (as acid) for mixtures of esters	European union (1223/2009)	This regulation sets the framework for cosmetic products in the EU. The EU limits the concentration of certain parabens in cosmetics. For example, propylparaben and butylparaben are restricted to 0.19% when used individually or in combination. Some parabens such as isopropylparaben, isobutylparaben, phenylparaben, benzylparaben, and pentylparaben, are banned due to safety concerns.	[4]
**USA**	0.4% for a single paraben	US-FDA	The U.S. Food and Drug Administration (FDA) monitors the safety of parabens in cosmetics and requires that all cosmetics be safe for use. Parabens as cosmetic additives should not	[5]

	0.8% for a mixture of parabens		exceed a concentration of 0.4% for a single ester and 0.8% for a mixture	
**Japan**	1% single paraben<br>1% for a mixture of parabens	Japan's Pharmaceutical Affairs Law	The pharmaceutical affairs law and its related regulations define the requirements for cosmetic products in Japan, including labeling, safety standards, and ingredient restrictions.	[6]
**Canada**	No set regulations	Health Canada (Food and Drugs Act) and Persistence and Bioaccumulation Regulations of CEPA (Canada, 2000)	Methylparaben, propylparaben, butylparaben and iso-butylparaben, ethylparaben, iso-propylparaben, and benzylparaben, were proposed not to meet the criteria for regulation as they are not entering the environment in a quantity or concentration or under conditions that constitute or may constitute a danger. All seven substances in this assessment were identified as having low potential to be causing ecological harm	[7,8]
**Denmark**	0.4% (as acid) for single ester,<br>0.8% (as acid) for mixtures of esters	Danish Ministry of the Environment. Statutory Order amending the Statutory Order on cosmetic products	Propylparaben, butylparaben, isopropylparaben, isobutylparaben and salts thereof must not be used in products for children under 3 years	[9]
**South Africa**	The combined concentration of propylparaben and butylparaben is restricted to 0.19%	South African Health Products Regulatory Authority (SAHPRA), No specific regulation	South Africa imposes limits on the concentration of parabens in cosmetic products similar to the EU	[4]

References

1. Tekin, Z.; Karlıdağ, N.E.; Özdoğan, N.; Koçoğlu, E.S.; Bakırdere, S. Dispersive Solid Phase Extraction Based on Reduced Graphene Oxide Modified Fe3O4 Nanocomposite for Trace Determination of Parabens in Rock, Soil, Moss, Seaweed, Feces, and Water Samples from Horseshoe and Faure Islands. *J Hazard Mater* **2022**, *426*, 127819.
2. Becerra-Herrera, M.; Miranda, V.; Arismendi, D.; Richter, P. Chemometric Optimization of the Extraction and Derivatization of Parabens for Their Determination in Water Samples by Rotating-Disk Sorptive Extraction and Gas Chromatography Mass Spectrometry. *Talanta* **2018**, *176*, 551–557.
3. Jiang, Y.; Bian, X.; Zhang, M.; Zhang, H.; Yu, K.; Kan, G.; Feng, Y.; Wang, X.; Song, D.; Jiang, J. Application of Dispersive Liquid–Liquid Extraction Followed by Rapid and Direct Mass Spectrometry Analysis to Evaluate Parabens in High Salinity Water Sample. *Microchemical Journal* **2023**, *194*, 109286.
4. UNION, P. Regulation (EC) No 1223/2009 of the European Parliament and of the Council. *Official Journal of the European Union L* **2009**, *342*, 59.

5. Nowak, K.; Ratajczak–Wrona, W.; Górska, M.; Jabłońska, E. Parabens and Their Effects on the Endocrine System. *Mol Cell Endocrinol* **2018**, *474*, 238–251.
6. Mishra, L.; Kurmi, B. Das Cosmetics Regulations and Standardization Guidelines. *Pharmaspire* **2023**, *15*, 137–150.
7. Pollock, T.; Karthikeyan, S.; Walker, M.; Werry, K.; St-Amand, A. Trends in Environmental Chemical Concentrations in the Canadian Population: Biomonitoring Data from the Canadian Health Measures Survey 2007–2017. *Environ Int* **2021**, *155*, 106678.
8. Khan, N.S. Childhood and Adolescent Exposure to Chemicals Found in Personal Care Products. **2023**.
9. Cabaleiro, N.; De La Calle, I.; Bendicho, C.; Lavilla, I. An Overview of Sample Preparation for the Determination of Parabens in Cosmetics. *TrAC Trends in Analytical Chemistry* **2014**, *57*, 34–46.
